# Phenotyping and clinical utility of phagocytic polyploid giant cancer macrophages in blood

**DOI:** 10.1016/j.canlet.2025.218007

**Published:** 2025-09-03

**Authors:** Daniel L. Adams, Massimo Cristofanilli, Steven H. Lin, Raymond C. Bergan, Thai H. Ho, Jeffrey R. Marks, Stuart S. Martin, Martin J. Edelman, Saranya Chumsri, Elizabeth J. Hager, Cha-Mei Tang, Susan Tsai, R. Katherine Alpaugh

**Affiliations:** a Creatv MicroTech, Inc., 9 Deer Park Dr., Monmouth Junction, NJ, 08852, USA; b Department of Medicine, Weill Cornell Medicine, New York Presbyterian Hospital, New York, NY, 10021, USA; c Northwestern University, Robert H Lurie Cancer Center, 303 E Superior, Chicago, IL, 60611, USA; d MD Anderson Cancer Center, Thoracic and Cardiovascular Surgery, 1515 Holcombe Blvd, Houston, TX, 77030, USA; e Oregon Health and Science University, 3303 SW Bond Ave., Portland, OR, 97239, USA; f Stony Brook University, Stoney Brook Cancer Center, 101 Nicolls Rd, Stony Brook, NY, 11794-7263, USA; g Mayo Clinic Arizona, 13400 East Shea Blvd., Scottsdale, AZ, 85054, USA; h Medical University of South Carolina, 86 Jonathan Lucas St, Charleston, SC, 29425, USA; i Duke University Medical Center, Department of Surgery, Division of Surgical Sciences, Durham, NC, 27710, USA; j University of Maryland Baltimore, Greenebaum Cancer Center, Baltimore, MD, 21136, USA; k Fox Chase Cancer Center, Protocol Support Laboratory, 333 Cottman Ave., Philadelphia, PA, 19111, USA; l Mayo Clinic Cancer Center, 4500 San Pablo Rd., Jacksonville, FL, 32224, USA; m Frederick National Laboratory for Cancer Research, National Cancer Institute, Biological Testing Branch, Frederick, MD, 21702, USA; n Creatv MicroTech, Inc., 9900 Belward Campus Dr., Rockville, MD, 20850, USA; o Medical College of Wisconsin, Milwaukee, WI, 53226, USA; p Ohio State University, Wexner Medical Center, 2050 Kenny Road, Columbus, OH, 43221, USA

## Abstract

Historically, polyploid giant cancer cells (PGCCs) within tumors have been ignored as superfluous inflammatory refuse with no intrinsic clinical or biological relevance. However recently, multiple studies have described the existence PGCCs in solid tumor masses that appear to correlate with tumor progression, and can also appear in blood circulation as cancer associated macrophage like cells (CAMLs). In an effort to understand the clinical and biological role of CAMLs (i.e. PGCCs in circulation), we initiated a multi-institutional 2 year prospective study of patients in an array of solid tumors (n = 293; breast, prostate, esophageal, lung, pancreas, or renal cell carcinoma), finding that CAMLs significantly correlate with progression and disease spread. We further evaluated the biological traits of CAMLs isolated from patients, identifying abnormal cellular characteristics including self-renewing proliferation, proangiogenic stem cell biomarkers, with overlapping myeloid, epithelial and endothelial characteristics. Here we report that CAMLs are highly indicative of disease progression in all cancer stages and appear to mimic phenotypes associated with metastatic niche initiation (i.e. traversing blood as self-renewing multipotent myeloid cells).

## 1. Introduction

The seed and foreign soil model of tumor spread theorizes that primary tumor cells foreign to metastatic sites initiate pre-metastatic niches (PMNs) by prompting pro-tumorigenic microenvironments in preparation for seed cells [1-5]. Circulating tumor cells (CTCs) derived from tumor masses have long been the focus of cancer research as they are the “seed” cells [3,5,6] which causes the initial tumor metastasis. However, we now understand that recruited myeloid derived progenitor cells (MPCs) can act as initiators of the “soil” prior to seeding [1-5,7]. The presence of cancer fosters MPC (i.e. CD14^+^, CD34^+^, VEGFR1/2+) recruitment and transformation from normal hematopoietic stem cells (HSCs) to tumor modified HSCs through a partially understood signaling mechanism involving chemokine and adrenergic receptors [1-5,7-14]. Cancer transformed HSCs then home to and initiates auxiliary PMNs prior to CTC seeding [1-5,7,9]. However, the connection between cancer and its’ ability to transform MPCs into pro-tumorigenic PMN initiators remains elusive [1-5,7]. Further, it is unknown why transformed MPCs recruit to preferential organ sites or how CTCs then home to the same sites after initiation [1-5,7]. This ability of MPCs and CTCs to separately and temporally colocalize to the same sites within an organ indicates an unknown orchestration of cellular movement originating at a primary tumor, through circulation and to PMNs [1-5,7]. Thus, the migratory process of PMN initiators occurs on a microscopic cellular level prior to visible spread of cancer (i.e. in local disease) becoming increasingly evident only after disease macroscopically appears at distant sites (i.e. non-local/metastatic disease) [1-5,7].

It has largely been assumed that the orchestration of the PMN and metastatic seeding revolves around the tumor’s ability to signal MPCs to initiate terraforming of foreign sites [3-6,9,15]. However, the process where normal MPCs from bone marrow are transformed by cancer cells and eventually initiate PMNs [1-5] via passage in the circulation has not been identified [1-6]. Recently, evidence has demonstrated that Polyploid Giant Cancer Cells (PGCCs) isolated from primary tumors appear to contain many of the attributes associated with a soil initiating cells [6,15-33] i.e. 1) immense biological complexity [6,15,19,21,25,26,34], 2) presence at primary tumor sites in premalignant legions, early stage and late stage carcinomas [6,15,16,18,19,21,23-26,34-36], 3) found traversing the blood and lymphatic circulation [4,6,15,17,20,22,35,37-39], 4) possessing multipotent niche forming capabilities [4,7,11,14,15,18,19,26], and 5) able to survive a foreign microenvironment [1-5,26,34]. Despite evidence that MPCs differentiate into PGCCs in the presence of solid tumors [1-5,7,11,17-19,21,24-26,28,30-34,40] and that PGCCs can be found in circulation bound to CTCs, studies have not evaluated circulating PGCCs relationship to cancer progression.

Despite PGCCs being found within most tumor masses exhibiting highly aberrant behavior, compared to that of typical mammalian cells (i.e. endoreplication, meiotic-like division, and cell fusion) they are typically ignored as inconsequential biological refuse [1-5,7,11,17-19,21,24-29,34,39,40]. PGCCs are biologically complex and have been shown to have stem cell qualities with increased tumorigenicity potential [11,18,19,21,24-26,34]. Further, PGCCs have been found proliferating *in vitro*, fusing with tumor cells, mimicking endothelial vasculature and providing cancer resistance/escape mechanisms during chemotherapeutic and radiotherapeutic regimes [18-21,24-29,34,37,41,42]. In inflammatory states, such as cancer, myeloid biased HSCs can give direct rise to giant polyploids with multipotent stem cell phenotypes and proangiogenic niche forming capabilities after rapidly egressing across endothelial layers from bone marrow niches [1-3,5,15,18,19,21,23,24,26,34,37,40,42]. PGCCs are found in all stages of solid cancer, negatively correlate with survival, and accumulate in metastatic organs (i.e. lung, bone, and liver), which have also been found in circulation defined as cancer associated macrophage-like cells (CAMLs) [6,15]. CAMLs appear in the circulation of most patients with solid tumors, including stage 0 (e.g. breast ductal carcinoma in situ (DCIS), but are rare in benign conditions and have not been identified in healthy individuals [6,15,17,35]. CAMLs are a myeloid derived phagocytic cPGCC defined by their specific presence in the circulation of malignant disease (i.e. cancer associated), CD14^+^ expression (i.e. myeloid derived), phagocytosis of cancer debris and/or engulfment of intact cancer cells (i.e. macrophage-like), with 7 μm having been previously evaluated and shown to allow the consistent capture of CAMLs and CTCs, while removing >99 % of RBCs and WBCs [6,15,17,35,43,44]. However, despite the parallels between CAMLs and tissue PGCCs, and the fact that CAMLs originate from primary tumors; there have been no studies into the clinical and biological relationship of CAMLs to cancer progression and spread [27,29,45,46].

## 2. Methods

### 2.1. Blood sample collection

This multi-institutional study consisted of 293 blinded anonymized cancer patient peripheral blood samples supplied through a collaboration agreement with Fox Chase Cancer Center, University of Maryland Baltimore, Duke University, Northwestern University, Medical College of Wisconsin, Mayo Cancer Clinic, MD Anderson Cancer Center, Oregon Health and Science University, and the National Institutes of Health all according to the local IRB approval and with written informed consent. Study group characteristics can be found in Supplementary Table S1. Blood samples (9–18 mL) were collected into CellSave preservative tubes^™^ (Menarini Silicon Biosystems), or heparinized vacutainers (for culture experiments). In addition, a total of 51 healthy volunteer blood samples were collected in CellSave^™^ preservative tubes, or heparinized vacutainers (for culture experiments), with written informed consent and IRB approval by Western IRB. Healthy volunteer samples were procured on a voluntarily basis with no selection process, outside standard exclusion criteria, as such all samples were considered from individuals in normal health.

### 2.2. CellSieve^™^ low-flow microfiltration procedure

Blood was processed by the CellSieve^™^ microfiltration technique at the 5 institutions as previously described [15,47,48]. Briefly, the microfiltration assay isolates circulating cells from whole peripheral blood based on size exclusion, >7 μm. CAMLs and CTCs are then stained and identified using the phenotypic expression of DAPI, CD45, CD14, and the Cytokeratins 8, 18, 19 (Figs. 2 and 3) using pre-established criteria.

### 2.3. Identification and enumeration of cells with large multilobed nuclei

For this study, CAMLs were morphologically identified using pre-established criteria, i.e. a single cell with an enlarged polyploid nuclear profile (>15 μm in diameter) with the nuclei surrounded by a larger cytoplasmic signal (25–350 μm in length), Fig. 2, Supplementary Figs. S2 and S3. After filtering and processing samples according to above protocols an Olympus BX54WI Fluorescent microscope with a Carl Zeiss AxioCam was used to image all CAMLs and CTCs. Exposures were preset for each individual flour for equal marker comparison [15,48]. A Zen2011 Blue (Carl Zeiss) was used to process the images. To measure sizes of cells, Zen2011 Blue (Carl Zeiss) software was pre-calibrated by the manufacturer. The automatic measurement software was used to find the length of CAMLs, CTCs and white blood cells (Supplementary Figs. S1 and S2).

### 2.4. Antibodies used

FITC labeled Cytokeratin 8, 18, & 19 cocktail [15], PE labeled EpCAM cocktail [15], Cy5 labeled CD45 cocktail [15,47,48], Vimentin (ebioscience),CD11c (ebioscience), CD31 (Miltenyi), CD34 (ebioscience), CD146 (ebioscience), (ebioscience), CD68 (Biolegend), androgen receptor (Cell Signaling), PSMA (Biolegend), CXCR4(ThermoFisher), CD202 (TIE2) (Biolegend), c-MET (Cell Signalingr), E-cadherin (ebioscience), CD105 (ebioscience), PDL1 [49], CCR5 (R&D systems), RAD50 (ThermoFisher), alpha tubulin (Cell Signaling), CD44 (ThermoFisher), CD24 (ThermoFisher), CD61 (ebioscience), and CD41 (ebioscience), CD38 (ebioscience), beta-2-adrenergic receptor (Cell Signaling),CD163 (Biolegend), VEGFR-1 (R&D Systems), CD133 (Miltenyi), CD144 (ebioscience), VEGFR2 (R&D Systems), and CD11b (ebioscience).

### 2.5. QUAS-R quenching and restaining

After initial identification and imaging of CAMLs, fluorescence was quenched and samples were restained with an additional antibody panel sets (vimentin/CD146/CD144, CD14/CD11b/CD41, CD11c/CD68/TIE2, or CD34/CD41/CD61) using the QUAS-R (Quench, Underivatize, Amine-Strip and Restain) technique, as previously described [48,49]. Briefly, after CAMLs are imaged and marked, cells on filters are subjected to sequential set of chemical treatments. Filters are demounted in PBS, placed into a reaction well, washed with 1X PBS, incubated with quenching solution and Tris for 1 h. After quenching, filters are washed, blocked with 1XPBS+20 %FBS+0.1 mg/mL whole mouse IgG (Jackson ImmunoResearch) and incubated with a secondary antibody panel and mounted. Samples were oriented and previously imaged CAMLs were relocated using a Zen2011 Blue (Carl Zeiss) mark and find software. After secondary restaining, the QUAS-R steps are repeated and samples restained with a third panel. After tertiary restaining, QUAS-R steps are repeated with a fourth antibody panel. Samples were not quenched past the fourth panel.

### 2.6. CEP17 FISH probe on CAMLs

PathVysion CEP17 DNA Probe Kit (Abbott) was supplied by Abbott Molecular Inc. CAML positive samples on the microfiltration assay were imaged, as above. Identified CAMLs were FISH probed directly on the filter as previously described [15]. In each case the x/y/z placement of cells were marked by etching the sample substrate and placement recorded using Zen2011 Blue (Carl Zeiss) [15,48,49]. Briefly, samples were demounted in 2X SSC and a protease solution for 20 min at 37 °C. Samples were washed in 2X SSC, dried, placed in denaturing solution, washed with 70 %–85 %–100 % ethanol and dried. 10 μL of CEP17 FISH probe was added, sealed with rubber cement and incubated overnight. Samples were washed, dried at room temp and mounted in Fluoromount-DAPI (Southern Biotech). CAMLs were imaged on an Olympus BX54WI Fluorescent microscope (Supplementary Figure S2).

### 2.7. Culture and expansion of cells

Heparinized blood specimens from cancer patients (n = 150) were collected at various cancer centers (ClinicalTrials.gov Identifier: NCT00900198), arriving within 16–28 h of collection. Patients were not receiving active systemic treatment, but were previously treated for malignancy. CAMLs were collected from whole peripheral blood by simple filtration as previously described (*38, 41, 46–47*). Filters were placed upside down in a plate and purified cells were cultured in medium composed of a modified DMEM/F15 (50:50), 5 % FBS; and 1 ng/ mL EGF, VEGF, PDGF, FGF basic, and IGF1; as previously described [21,25,26,47,50]. CAML cultures were proliferated until confluence or senescence (Fig. 4 and Supplementary Fig. S5).

### 2.8. Statistical methods

Analyses were made with MATLAB R2021A using the counts from all patient samples. For overall survival (OS) analysis, significance of Kaplan Meier plots were determined by log-rank, and OS was defined as the interval between blood sample draw to date of death or censored based on date of last known follow up visit. Progression free survival (PFS) was determined based on radiographic or clinical confirmation of disease spread or tumor growth.

## 3. Results

**Fig. 1.**
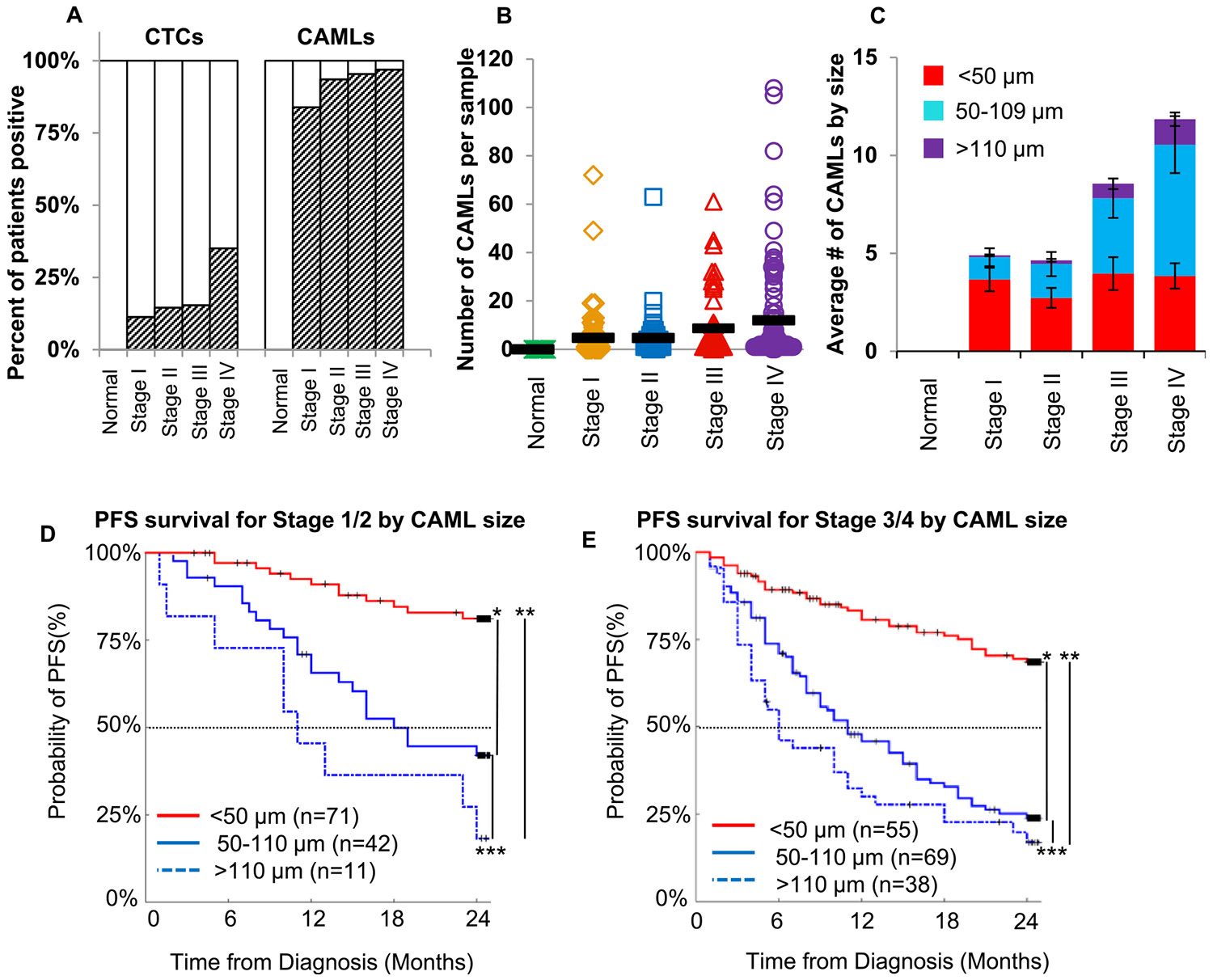
Clinical prevalence of CAMLs in solid malignancies. **A.** Prevalence of CAMLs and CTCs in 7.5 mL blood samples (n = 293 cancer patients and n = 51 healthy normal controls). **B.** Number of CAMLs in each 7.5 mL patient sample (Black bar = average). **C.** Average number of CAMLs classified by 3 sizes based on the observed biomodal distribution (Supplementary Fig. 1). **D and E.** CAMLs are present in early and late stage disease. Their larger diameter is an indicator of disease progression (**D** *p < 0.0001, ** p < 0.0001, ***p = 0.1604) (**E** *p < 0.001,** p < 0.001, ***p = 0.2973). Stage1/2 0-<50 μm vs 50–110 HR = 4.8 (95 %CI 2.4–9.9), <50 μm vs > 110 HR = 56.9 (95 %CI 12.9–251.1), 50–110 vs > 110+ HR = 2.2 (95 %CI 0.9–5.5). Stage 3/4 < 50 μm vs 50–110 HR = 3.1 (95 %CI 2.0–5.0), <50 μm vs > 110 HR = 3.6 (95 %CI 1.9–6.6), 50–110 vs > 110 HR = 1.1 (95 %CI 0.7–1.8). Unstaged patients were not included this analysis (n = 7). Number at risk can be found in Supplementary Tables 1, 2 and 3.

**Fig. 2.**
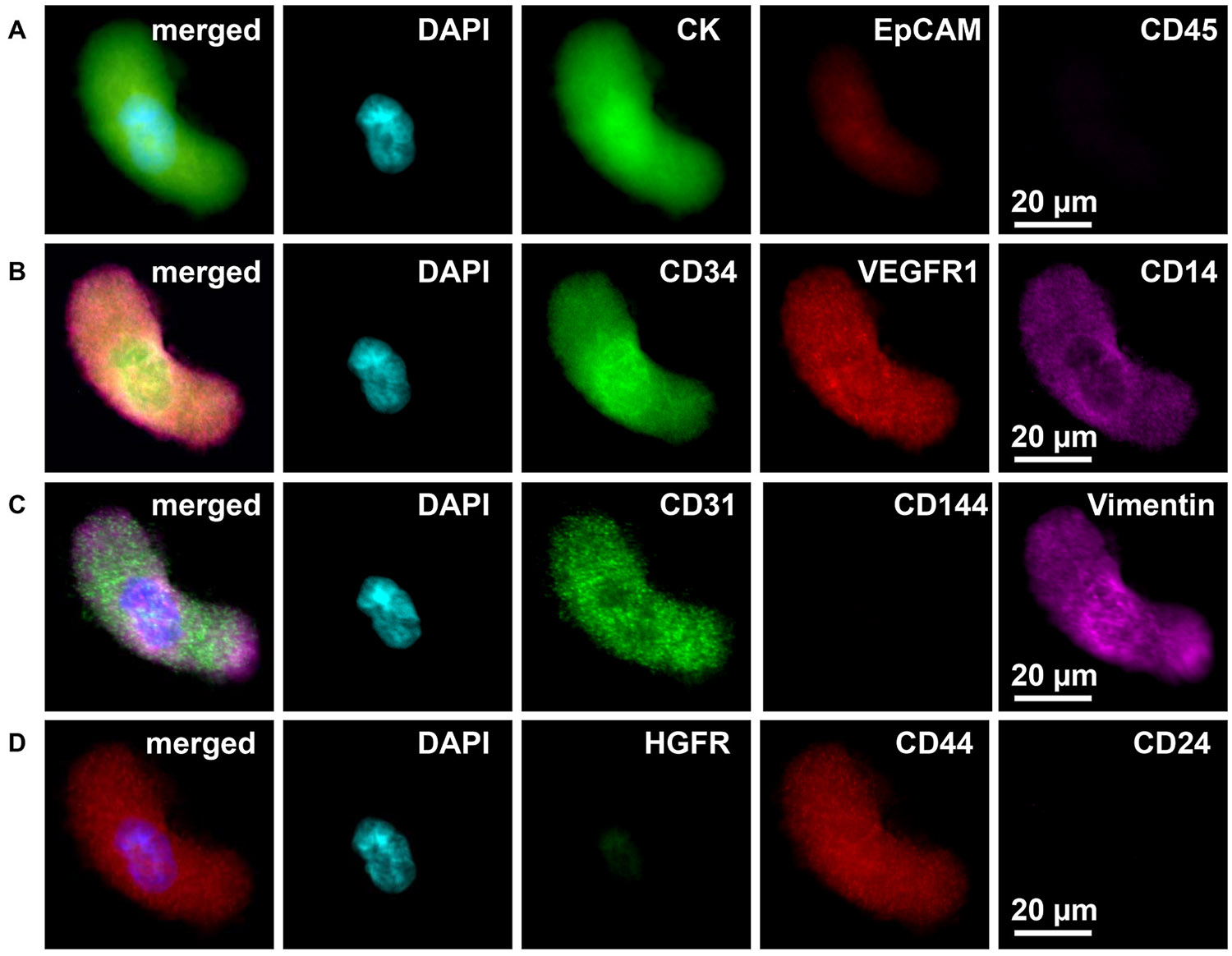
Sequential restaining of an individual CAML by fluor quenching using QUAS-R for cell identification and subtyping. **A.** Classical CTC phenotype: Cytokeratin (CK) and EpCAM show the epithelial characteristics on a CAML, while absence of CD45 shows the CAML is not a leukocyte. **B.** Pre-metastatic niche initiator (PMN) phenotype: CD14 (pan-myeloid) defines the CAML as myeloid while dual expression of CD34 (stem cell) and VEGFR1 (angiogenic) is an established PMN phenotype^2^. **C.** Angiogenic/Stem cell phenotype: CD31 (angiogenic) and CD144 (angiogenic) are adhesion molecules commonly found on stem cells and vascular junctions, while Vimentin (mesenchymal) along with CK indicates the CAML is both mesenchymal and epithelial. **D.** Stem Cell phenotype: CD44 (Stem cell) without CD24 (B-cell) is a common identifier of cancer stem cells, while HGFR (proto-oncogene) is a common signaling receptor in cancer. Square = 60um.

We assessed a large multi-institutional pilot study of patients (n = 293) with variety of solid malignancies to determine if CAMLs presented prior to or after metastatic formation, as well as their relationship to clinical progression [1-5]. We initiated a multi-year prospective study to evaluate the clinical presentation of CAMLs, and CTCs, across numerous epithelial cancers [breast (n = 59), prostate (n = 52), lung (n = 59), pancreatic (n = 59), esophageal (n = 27), and renal cell cancers (n = 37)] (Supplementary Tables S1, S2, S3 S4, S5 and S6). A low pressure filtration system using array patterned microfilters isolated CAMLs from 7.5 mL whole peripheral blood. CAMLs were identified by an enlarged size (25–350 μm) (Supplementary Figure S1), polyploid nucleus and expression of epithelial (cytokeratin), leukocyte (CD45), and/or myeloid (CD14) phenotypes. After CAMLs were identified using these biomarkers and an enlarged size, CAMLs polyploid nucleus was then measured by a CEP17 FISH with all pre-identified CAMLs having a ≥6N ploidy status (6N–68N), as to remove the possibility of normal dividing cells 4N cells (Figs. 1 and 2, Supplementary Fig. S2). In the 293 patients tested, CAMLs were found in 93 % of patient samples, including pre-metastatic patients, Stage I (84 %), Stage II (94 %) Stage III (95 %) and Stage IV (97 %) patients (Fig. 1) but absent in all healthy controls.

Since increased cell size/ploidy status of PGCCs within tumors is known to be associated with grade and cancer progression [16,18,19,22-25,27,29,34,36,45,46], we assessed if a similar relationship between the sizes of CAMLs, as a proxy for polyploid number (N), and disease stage. We grouped patients into three size classes (<50 μm, 50–110 μm and >110 μm) based on an observed and modeled bimodal pattern in the frequency of CAML size/ploidy (Fig. 1 and Supplementary Fig. S1). We observed that CAMLs <50 μm were found equally in all stages and subtypes of diseases, suggesting a constant flux of this size class of smaller CAMLs into the circulatory system of patients with solid tumors as a result of an inflammatory response to cancer. Additionally, both the 50–110 μm and the >110 μm sizes appeared to exponentially increase with stage of disease (Fig. 1C and Supplementary Fig. S1), suggesting cellular aberration increases as the cancer spreads. Further, at 24 months a significant correlation to progression free survival (PFS) in patients was found, with the larger CAML sizes 50–110 μm and >110 μm having significantly poorer clinical outcomes (Fig. 1D and E, Supplementary Tables S1, S2 and S3).

In comparison, CTCs appear primarily in patients with metastatic disease (i.e. macroscopic secondary/tertiary legions), long after the presence of non-local microscopic disease (Fig. 1A) [1,3-6,15,35]. While focusing on tumor initiating cells (e.g. CTCs) purified from patients may predict treatment response in advanced metastatic disease, CAMLs appear to predict patient’s treatment response in localized disease where curative intent is still possible. Further, while our enumeration of CTCs did show correlation with PFS, the appearance of CTCs were largely undetectable in non-metastatic disease (Stage I, II and III), occurring only after disease had spread to other organs (Fig. 1A). This data suggests that unlike CTCs which are associated with late stage disease, CAMLs are clinically present in both early localized or later non-local disease and are highly correlated with eventual tumor progression and metastasis. CAMLs also appeared far lower in treatment naïve metastatic patients averaging 3.57 CAMLs/samples in treated naïve patients versus 17.4 in patients receiving chemotherapy, 10.37 in patients receiving hormone therapy and 12.75 in patients receiving targeted therapy (Supplementary Table S6). Further, while there was little relationship between CAMLs and site of metastases, with a possible exception of adrenal metastatic spread, patients with more than one organ of metastatic involvement had a 214 % increase in CAML number than patients with only 1 organ with metastatic spread (ANOVA p = 0.0201). While only a pilot study on an array of different cancer subtypes, CAMLs appear to correlate with aggressiveness of localized cancers and may be predictive of a cancers ability to spread to multiple organs after metastatic spread, suggesting a relationship with cancer pathogenesis.

**Fig. 3.**
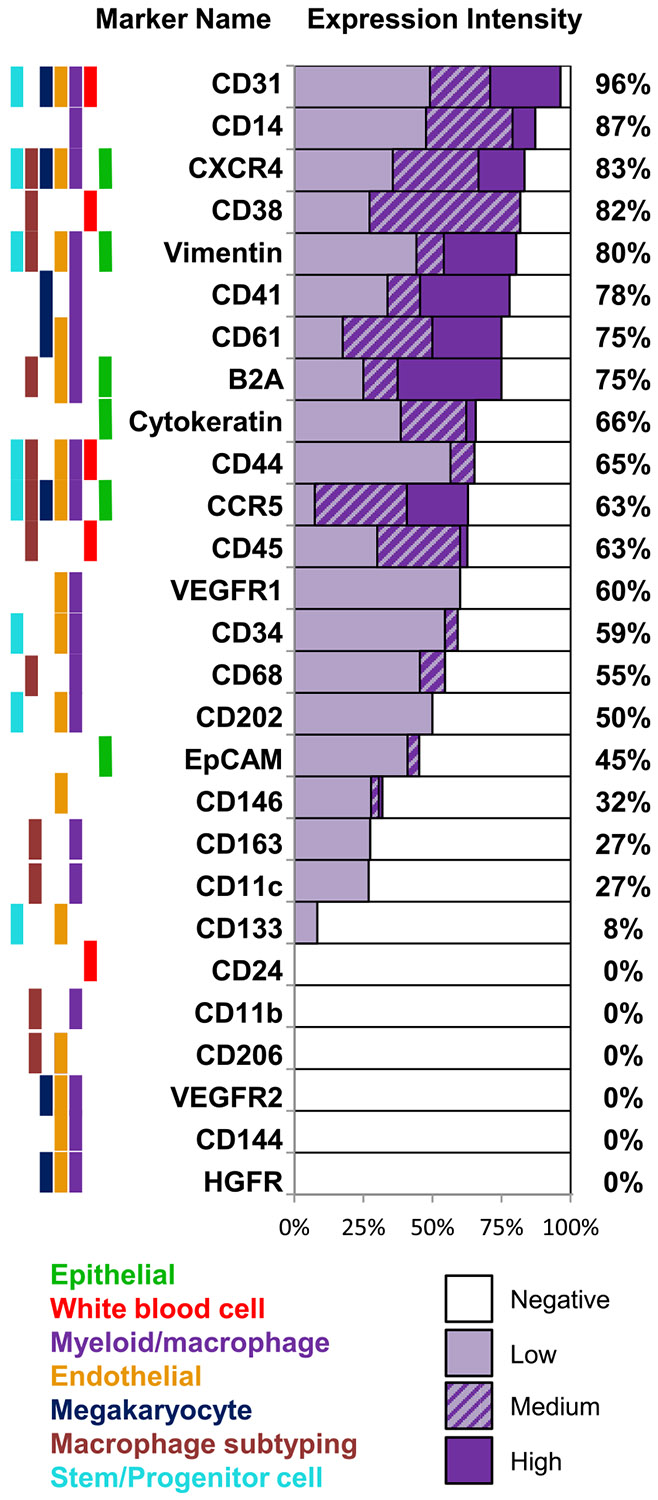
Identifying and Subtyping CAMLs based on presence and intensity of cell differentiation markers. Colored bar code (left) represents the biological utility of the markers used in immunophenotyping human cell populations. Purple bars (center) are the percent of tested CAMLs that were negative, low, medium, or high expressing for each marker screened. Total positivity is shown to the right of the bar chart. Markers were screened against 1118 CAMLs from 93 different patients with cancer.

PGCCs and normal giant ployploids are derived from MPCs which can harbor multipotent stem cell phenotypes, reside in HSC niches, rapidly egress across endothelial layers and express proangiogenic niche forming capabilities [4,7,11,14,18-23,26,34,36,38-40]. Unlike tissue PGCCs, phenotyping their CAML counterpart in circulation is difficult because of low abundance (i.e. averaging ~1 CAML/mL blood) and the heterogeneous nature of CAMLs, including an expression of overlapping epithelial (cytokeratin and EpCAM), endothelial (CD146) and macrophage (CD14 and CD11c) phenotypes [6,15,35,37]. We established a technique to mass screen numerous phenotypic protein markers on individual cells as to properly subtype individual CAMLs. Quench Underivatize Amine Strip and Restaining (QUAS-R) [48] has been shown to nondestructively remove fluorescence from stained samples allowing restaining of >12 additional characterization markers (Fig. 2) for each cell. CAMLs were initially identified via DAPI, cytokeratin, and CD45; then sequentially restained with 27 additional myeloid, leukocyte, megakaryocyte, epithelial, endothelial, progenitor/stem, and motility markers (Figs. 2 and 3). Most CAMLs were found to express levels of CD31 (myeloid/endothelial), and commonly coexpressed cytokeratin (epithelial), CD14 (myeloid) and CD38 (white blood cell). However, while CAMLs contained clear myeloid lineage (CD14), no other cell definable differentiation marker was universal. Further, CAMLs presented with numerous phenotypes which do not appear to match our understanding of classical cellular differentiation (i.e. coexpression of CD45 [leukocyte] and cytokeratin [epithelial], CD11c [macrophage] and CD41 [megakaryocyte], CD146 [endothelial] and CD41/CD61 [megakaryocyte], CD41/CD61 [megakaryocyte] and CD68/CD163 [scavenger macrophage]) (Fig. 3). Combined, these data show CAMLs are myeloid derived cells that possess many phenotypic attributes associated with stem cell and proangiogenic phenotypes [1,14,19,21,26,34,40,42].

Myeloid biased HSCs (CD34+/CD41+/CD14+ cells) egress from HSC bone marrow niches through a number of mobilization receptors (CXCR4, CCR5 and catecholaminergic neurotransmitters) [1,4,7-14,19,20,23,38-40,42]. Our analysis of commonly known overlapping myeloid biased HSC characteristics (Fig. 3) suggests that CAMLs express similar stem cell traits (e.g. CD34, CD41, CD31, VEGFR1), myeloid traits (CD14 and CD41) and these same receptors have been associated with egress via endothelial migration (CXCR4, CCR5 and B2A) [1,4,7-14,19,20,23,38-40,42]. Consistent with a motile phenotype, CAMLs were found to express recognized markers of highly motile cells (CXCR4, CCR5, Vimentin, alpha-tubulin) and biologically active forms of cancer associated mobilization receptors (CCR5 and beta-2-adrenergic receptor) (Figs. 2 and 3 and Supplementary Fig. S3). Interestingly, cells with myeloid HSCs traits (CD14+/CD34+/VEGFR1+) have recently been identified as the initiators of PMNs, recruiting to pre-metastatic tissues, initiating neo-angiogenesis, promoting tumor adherence and enhancing tumor growth [1,2,5,7]. This same myeloid HSC phenotype (CD14+/CD34+/VEGFR1+) was identified in 25 % of CAMLs tested (Figs. 2 and 3) and the proangiogenic phenotype (CD31+/TIE-2+) was found in another 45 % of CAMLs. While a relationship between a primary tumor and myeloid HSCs is required for PMN formation, the myeloid biased HSC cell type that egresses from HSC niches and transforms into a proangiogenic PMN initiator has not been identified. However, these data show that CAMLs express myeloid stem cell and proangiogenic phenotypes which appear to show biological activity to the HSC mobilization receptors. This suggests that CAMLs are phenotypically similar to the circulating stage of a myeloid biased PGCC after egress from a niche, likely as a result of transformation by inflammation due to presence of a solid tumor.

**Fig. 4.**
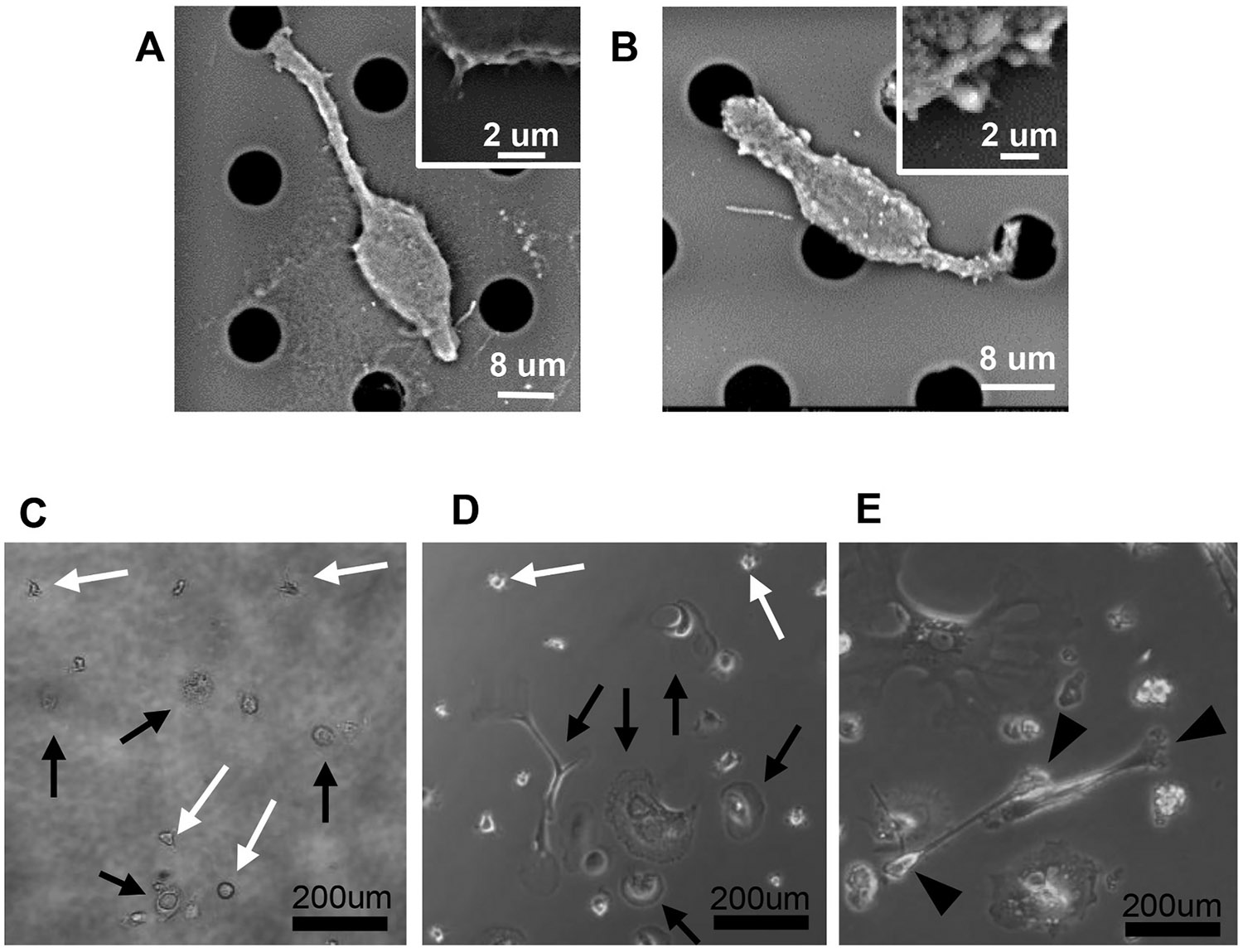
SEM of CAMLs with pseudopodia like protrusions or blebbing structures, and the proliferation of CAMLS *in vitro*. **A.** SEM of a CAML isolated on a microfilter with multiple small pseudopodia like protrusions emanating from the cell membrane and a zoomed in image of one of the protrusions (upper right). **B.** SEM of a CAML isolated on a microfilter with blebbing structures of various sizes breaking off for the primary cell mass and a zoomed in image of the blebs (upper right). **C.** After filtration, isolated blood cells were placed into culture flask and multiple CAMLs (black arrows) were observed. **D.** The same CAMLs were re-imaged after 5–21 days CAMLs finding CAMLs had enlarged, with many forming large pseudopodia and auxillary structures (Supplementary Fig. 5). **E.** The same CAMLs were re-imaged again after 45 days, when CAMLs then began to proliferate by cellular budding with new cells forming at the tips of the protrusions (block black arrows). The daughter cells eventually budded from the main structure and continued to proliferate in a similar fashion. CAMLs commonly reached 50–75 % confluency and after 45–180 days of proliferation. (white arrows = white blood cell contaminates).

Recently PGCCs from solid tumors (e.g. glioblastoma, ovarian, breast and prostate) have been shown to proliferate *in vitro*, possess stem cell like phenotypes [19,21,25,26,34,41], increased tumorigenic potential, and preferentially arrest in lymph nodes, lung, liver and brain capillaries [11,16,19,21,22,25,26,34,41,51]. Under scanning electron microscopy (SEM), CAMLs were observed with two distinct extracellular mechanisms which match with descriptions of *in vitro* PGCC proliferation, including blebbing/budding of the cell membrane and pseudopodia formation (Fig. 4A and B and Supplementary Fig. S4). To determine proliferative capability, CAMLs were purified from a randomly selected cohort of cancer patient blood samples (n = 150) (Supplementary Figure S5) [47,50] and plated in modified DMEM media [25,26]. From 22 % of patient blood samples, but in none of the normal controls, CAMLs adhered to the plate with pseudopodia that spread and elongated, producing giant, pleiotropic, multinucleated cells (Fig. 4C-E). In contrast to our understanding of human mitotic division, but in agreement with studies on tissue derived PGCCs [21,25,26,34,41], CAMLs produced progeny in an asymmetrical meiotic-like fashion, forming as small circular blebs/buds along the surface of the primary giant cell and appearing as “satellite” cells (Supplementary Fig. S5). After 2–5 days, the original giant cell disintegrated and the smaller satellite cells enlarged, undergoing multiple rounds of expansion until confluence and/or senescence after 45–180 days. We then compared the consistency of key biomarkers from cultured CAMLs (13–40 days *in vitro*) against the biomarkers from non-cultured CAMLs (isolated directly from blood) using parallel blood samples (Supplementary Figs. S6 and S7), finding that cultured CAMLs retained the myeloid and stem cell traits (CD31, CD14 and CD44, Vimentin) but reduced epithelial and phagocytic traits (CD11c, CD68 and cytokeratin) (Fig. 2 and Supplementary Figure S6, S7 and S8). This loss in epithelial traits may be a result of phagocytosed material being expelled into the media from cells, or possibly the spread in ingested material between multiple CAML progeny as they expanded, though these hypotheses must be further vetted. Additionally, no development of enlarged cells was seen in match blood from normal healthy control samples. These data suggest that CAMLs are a cancer specific cell type transformed by the presence of solid tumors that circulate the vasculature with proangiogenic stem-like phenotypes that retain viability and the ability to rapidly proliferate *in vitro*.

## 4. Discussion

Normally occurring giant cells and HSCs have numerous overlapping features, i.e. niche forming receptors [7,11,14,19,24,40], egress from bone marrow niches [8-10,12,13], migrate to and through endothelial junctions by producing matrix metalloproteinases [4,20,38,39], and preferentially lodge in organs common to metastatic spread [16,17,21,25,34,51]. In cancer, PGCCs have these same attributes, but also exist in most solid tumors (a.k.a. polyploid giant cancer cells or monster cells) [19,21,23,24,26,34,36] and in the circulation (i.e. CAMLs) [6,15,17,27,29,37,45,46]. It has been previously described that CAMLs emanate from a patient’s primary mass in NSCLC and esophageal cancer (Supplementary Figure S9) [49], circulating through blood in early stage cancer [6,15,35] and can be found bound to CTCs [15]. Here, we looked to better evaluate CAMLs by tracking their biological and clinical utility in an array of cancer patients. In line with prior studies, we found that CAMLs could be found in 93 % of all cancer patients, including 84 % of Stage I disease. Further, we found that stratifying CAMLs based on their size/ploidy, i.e. larger then ≥50 μm, could stratify patients into those with poorer PFS and OS irregardless of disease stage. These data suggest that CAMLs represent a universal biomarker for solid tumors whose larger size is clinically associated with more aggressive disease. While larger validation studies are now required, these findings suggest that CAMLs act as a non-invasive blood based biomarker in local and advanced disease in an array of cancer subtypes.

In our observational studies of CAMLs, we found they possess many of the traits expressed by PMN initiating cells and suggest a new model between cancer modified myeloid biased HSCs (i.e. CAMLs) and the ability of cancer to spread [1-5]. Being that CAMLs are a PGCC subtype, and larger cells have mechanistic properties associated with preferential arrest in organs of metastatic preference (i.e. lung and liver) [11,16,20,22,24,34,51], it is interesting that circulating PGCCs have never been evaluated in the context of PMN formation. Along with their unusually large morphological attributes, we show that CAMLs have proangiogenic myeloid stem cell phenotypes and bind to CTCs in circulation [15]. Interestingly, these CAML characteristics could logically explain the ability to orchestrate temporal homing of PMN initiators along with CTCs to pre-established PMN sites by the fact that large cells are mechanistically prone to arresting in the same capillaries within organs based on their large size [51]. However, while the findings that the interactions between CTCs and CAMLs appear biologically and clinically relevant, further investigations regarding this interaction within the tumor microenvironment and role in the metastatic process requires further investigation.

Overall, we show that CAMLs possess a multi-pronged repertoire of attributes associated with PMN initiation while providing evidence of a consorted orchestration between CAMLs and CTC migration [15,28,30-33]. Here we further describe biological attributes of CAMLs as they relate to cancer including 1) presence at primary tumor sites including local and non-local disease, 2) highly motile cells which traverse the blood circulation 3) express proangiogenic multipotent stem cell phenotypes, and 5) are able to independently proliferate in foreign microenvironments. These data indicate that CAMLs have numerous biological attributes which are considered abnormal and are able to proliferate in an asymmetrical meiotic-like fashion (Fig. 4 and Supplementary Fig. S5). Further, while here we describe CAML expansion *in vitro* and within a culture flask, future studies should evaluate whether CAMLs can expand within mice models, within the tumor itself, or possibly at metastatic sites.

In studying the phenotypes of CAMLs to better elucidate the cellular origins of CAMLs, we found that CAMLs express a number of contrasting cellular receptors not typical in normal humans with CD31, CD14, CD41 and cytokeratin. While CD14, along with numerous other myeloid/marcrophage receptors (CD68, CDCD38 & CD31), was used to definitively identify CAMLs as myeloid origin, the presence of epithelial and endothelial receptors complicated any definitive cell subtyping. The coexpression of Cytokeratin and Vimentin, which is typically associated with a dual mesenchymal and epithelial phenotype, further convoluted the classification of these cells, though not surprising as phagocytic CAMLs are likely engorged on epithelial tumor proteins. While interesting findings, further mRNA, enzymatic and in-depth proteomic profiling of CAMLs must be evaluated to determine why some of the subcellular biomarker staining patterns within CAMLs do not appear aligned with normal cellular placement, such as CD45 and EpCAM appearing cytoplasmic instead of extracellular. While all antibodies used were purchased from well-established manufacturers, used within recommended concentrations for immunofluorescence, and screened against positive and negative cells lines, additional mRNA testing is needed to evaluate the causes of these irregular subcellular placements (Supplementary Table S7). This may include normal macrophage phagocytosis to within the cell cytoplasm, or possibly a tumor cell and macrophage fusion event, or some other cellular event that still needs to be elucidated [14,25,26,28,29,52]. Despite the complex biological variations we found in CAMLs, the clinical analysis was fairly definitive. Presence of CAMLs appears to be specific to active malignancy (93 % of all patients), as they were not found in any healthy controls (Fig. 1). We found that the numbers of CAMLs in patients were exponentially associated with stage of disease. Further, these data show that smaller low ploidy CAMLs appear common in almost all patients with active malignancy, in newly diagnosed untreated patients or patients undergoing therapy. In contrast, larger higher ploidy CAMLs seem to be associated to patients with more aggressive malignancies and are significantly associated with poorer outcomes. However, while a size-based analysis is a relatively straight forward approach, which appears to stratify most clinically relevant CAML sub-types, further analysis into the biological mechanisms of CAMLs, i.e. mitotic analysis, mRNA and in-depth molecular profiling, is now needed to better extrapolate the most important underlying biological mechanisms.

Combined these data present the largest and most in depth study of circulating PGCC, a common cell found in the circulation of cancer patients. These preliminary findings suggest that CAMLs might be an important clinical tool in the identification of cancer as well as in the early treatment of cancer. However, now that this initial evaluation of CAMLs has been done, future studies need to better compare each CAML phenotype and their association with tumor type, stage, progression, treatment and other clinical variables. Further, studies are also needed to expand the sample size, test specific refined populations, and conduct blinded multi-center validation experiments to validate these clinical findings. Overall, these initial clinical findings regarding CAMLs show promise with more defined cohort studies in specific treatment regimes needed to determine if better patient outcomes can be accomplished.

## Supplementary Material

Appendix A. Supplementary data

## Appendix A. Supplementary data

Supplementary data to this article can be found online at https://doi.org/10.1016/j.canlet.2025.218007.

## References

[1] Kaplan RN, Riba RD, Zacharoulis S, Bramley AH, Vincent L, Costa C, MacDonald DD, Jin DK, Shido K, Kerns SA, Zhu Z, Hicklin D, Wu Y, Port JL, Altorki N, Port ER, Ruggero D, Shmelkov SV, Jensen KK, Rafii S, Lyden D, VEGFR1-positive haematopoietic bone marrow progenitors initiate the pre-metastatic niche, Nature 438 (2005) 820–827. 16341007 10.1038/nature04186 PMC2945882

[2] Kim S, Takahashi H, Lin WW, Descargues P, Grivennikov S, Kim Y, Luo JL, Karin M, Carcinoma-produced factors activate myeloid cells through TLR2 to stimulate metastasis, Nature 457 (2009) 102–106. 19122641 10.1038/nature07623 PMC2746432

[3] Langley RR, Fidler IJ, The seed and soil hypothesis revisited–the role of tumor-stroma interactions in metastasis to different organs, International journal of cancer. Journal international du cancer 128 (2011) 2527–2535. 21365651 10.1002/ijc.26031 PMC3075088

[4] Psaila B, Lyden D, The metastatic niche: adapting the foreign soil, Nat. Rev. Cancer 9 (2009) 285–293. 19308068 10.1038/nrc2621 PMC3682494

[5] Wels J, Kaplan RN, Rafii S, Lyden D, Migratory neighbors and distant invaders: tumor-associated niche cells, Genes Dev. 22 (2008) 559–574. 18316475 10.1101/gad.1636908 PMC2731657

[6] Adams DL, Cristofanilli M, Detecting and monitoring circulating stromal cells from solid tumors using blood-based biopsies in the twenty-first century: have circulating stromal cells come of age? in: Cristofanilli M (Ed.), Liquid Biopsies in Solid Tumors Springer International Publishing, Cham, 2017, pp. 81–104.

[7] Casella I, Feccia T, Chelucci C, Samoggia P, Castelli G, Guerriero R, Parolini I, Petrucci E, Pelosi E, Morsilli O, Gabbianelli M, Testa U, Peschle C, Autocrine-paracrine VEGF loops potentiate the maturation of megakaryocytic precursors through Flt1 receptor, Blood 101 (2003) 1316–1323. 12406876 10.1182/blood-2002-07-2184

[8] Bronte V, Bria E, Interfering with CCL5/CCR5 at the Tumor-Stroma interface, Cancer Cell 29 (2016) 437–439. 27070698 10.1016/j.ccell.2016.03.019

[9] Chow MT, Luster AD, Chemokines in cancer, Cancer Immunol. Res 2 (2014) 1125–1131. 25480554 10.1158/2326-6066.CIR-14-0160 PMC4258879

[10] Cole SW, Sood AK, Molecular pathways: beta-adrenergic signaling in cancer, Clin. Cancer Res 18 (2012) 1201–1206. 22186256 10.1158/1078-0432.CCR-11-0641 PMC3294063

[11] Lefrancais E, Ortiz-Munoz G, Caudrillier A, Mallavia B, Liu F, Sayah DM, Thornton EE, Headley MB, David T, Coughlin SR, Krummel MF, Leavitt AD, Passegue E, Looney MR, The lung is a site of platelet biogenesis and a reservoir for haematopoietic progenitors, Nature 544 (2017) 105–109. 28329764 10.1038/nature21706 PMC5663284

[12] Perez-Sayans M, Somoza-Martin JM, Barros-Angueira F, Diz PG, Gandara Rey JM, Garcia-Garcia A, Beta-adrenergic receptors in cancer: therapeutic implications, Oncol. Res 19 (2010) 45–54. 21141740 10.3727/096504010x12828372551867

[13] Sloan EK, Priceman SJ, Cox BF, Yu S, Pimentel MA, Tangkanangnukul V, Arevalo JM, Morizono K, Karanikolas BD, Wu L, Sood AK, Cole SW, The sympathetic nervous system induces a metastatic switch in primary breast cancer, Cancer Res. 70 (2010) 7042–7052. 20823155 10.1158/0008-5472.CAN-10-0522 PMC2940980

[14] Woolthuis CM, Park CY, Hematopoietic stem/progenitor cell commitment to the megakaryocyte lineage, Blood 127 (2016) 1242–1248. 26787736 10.1182/blood-2015-07-607945 PMC5003506

[15] Adams DL, Martin SS, Alpaugh RK, Charpentier M, Tsai S, Bergan RC, Ogden IM, Catalona W, Chumsri S, Tang CM, Cristofanilli M, Circulating giant macrophages as a potential biomarker of solid tumors, Proc. Natl. Acad. Sci. U. S. A 111 (2014) 3514–3519. 24550495 10.1073/pnas.1320198111 PMC3948254

[16] Brill R, Halpern MM, The frequency of megakaryocytes in autopsy sections, Blood 3 (1948) 286–291. 18902576

[17] Hume R, West JT, Malmgren RA, Chu EA, Quantitative observations of circulating megakaryocytes in the blood of patients with cancer, N. Engl. J. Med 270 (1964) 111–117. 14067007 10.1056/NEJM196401162700301

[18] Illidge TM, Cragg MS, Fringes B, Olive P, Erenpreisa JA, Polyploid giant cells provide a survival mechanism for p53 mutant cells after DNA damage, Cell Biol. Int 24 (2000) 621–633. 10964452 10.1006/cbir.2000.0557

[19] Larsson LI, Cell Fusions: Regulation and Control, Cell Fusions: Regulation and Control, Springer Netherlands, Dordrecht, 2011.

[20] Papadimitriou JM, Kingston KJ, The locomotory behaviour of the multinucleate giant cells of foreign body reactions, J. Pathol 121 (1977) 27–36. 327038 10.1002/path.1711210105

[21] Qu Y, Zhang L, Rong Z, He T, Zhang S, Number of glioma polyploid giant cancer cells (PGCCs) associated with vasculogenic mimicry formation and tumor grade in human glioma, J. Exp. Clin. Cancer Res 32 (2013) 75. 24422894 10.1186/1756-9966-32-75 PMC4029228

[22] Soares FA, Increased numbers of pulmonary megakaryocytes in patients with arterial pulmonary tumour embolism and with lung metastases seen at necropsy, J. Clin. Pathol 45 (1992) 140–142. 1541694 10.1136/jcp.45.2.140 PMC495657

[23] Storchova Z, Pellman D, From polyploidy to aneuploidy, genome instability and cancer, Nat. Rev. Mol. Cell Biol 5 (2004) 45–54. 14708009 10.1038/nrm1276

[24] Winkelmann M, Pfitzer P, Schneider W, Significance of polyploidy in megakaryocytes and other cells in health and tumor disease, Klin. Wochenschr 65 (1987) 1115–1131. 3323647 10.1007/BF01734832

[25] Zhang D, Yang X, Yang Z, Fei F, Li S, Qu J, Zhang M, Li Y, Zhang X, Zhang S, Daughter cells and erythroid cells budding from PGCCs and their clinicopathological significances in colorectal cancer, J. Cancer 8 (2017) 469–478. 28261349 10.7150/jca.17012 PMC5332899

[26] Zhang S, Mercado-Uribe I, Xing Z, Sun B, Kuang J, Liu J, Generation of cancer stem-like cells through the formation of polyploid giant cancer cells, Oncogene 33 (2014) 116–128. 23524583 10.1038/onc.2013.96 PMC3844126

[27] Schmidt MJ, Naghdloo A, Prabakar RK, Kamal M, Cadaneanu R, Garraway IP, Lewis M, Aparicio A, Zurita-Saavedra A, Corn P, Kuhn P, Pienta KJ, Amend SR, Hicks J, Polyploid cancer cells reveal signatures of chemotherapy resistance, Oncogene 44 (2025) 439–449. 39578659 10.1038/s41388-024-03212-z PMC11810791

[28] Sutton TL, Patel RK, Anderson AN, Bowden SG, Whalen R, Giske NR, Wong MH, Circulating cells with macrophage-like characteristics in cancer: the importance of circulating neoplastic-immune hybrid cells in cancer, Cancers 14 (2022). 10.3390/cancers14163871 PMC9405966 36010865

[29] Wu TP, Li X, Ba S, Jones P, Hansel DE, Liu J, Meeting report: 1st international conference on polyploid giant cancer cells-biology, clinical applications, and the birth of a new field in cancer research, Cancer Lett. 612 (2025) 217447. 39793754 10.1016/j.canlet.2025.217447 PMC12186869

[30] Iwase T, Parikh A, Wenli D, Shen Y, Adams DL, Tang CM, Cohen EN, Reuben JM, Shrimanker TV, Chainitikun S, Kida K, Raghavendra AS, Sapon ME, Sahin O, James A, Sridhar N, Klopp AH, Tripathy D, Ueno NT, Circulating cancer-associated macrophage-like cells and macrophage-related cytokines in Obese patients with advanced breast cancer who undergo neoadjuvant chemotherapy, J. Cancer 15 (2024) 5855–5862. 39440056 10.7150/jca.89453 PMC11493001

[31] Jiao Y, Yu Y, Zheng M, Yan M, Wang J, Zhang Y, Zhang S, Dormant cancer cells and polyploid giant cancer cells: the roots of cancer recurrence and metastasis, Clin. Transl. Med 14 (2024) e1567. 38362620 10.1002/ctm2.1567 PMC10870057

[32] Krotofil M, Tota M, Siednienko J, Donizy P, Emerging paradigms in cancer metastasis: ghost mitochondria, vasculogenic mimicry, and polyploid giant cancer cells, Cancers (2024) 16. 10.3390/cancers16203539 PMC11506636 39456632

[33] Lopez-Collazo E, Hurtado-Navarro L, Cell fusion as a driver of metastasis: re-evaluating an old hypothesis in the age of cancer heterogeneity, Front. Immunol 16 (2025) 1524781. 39967663 10.3389/fimmu.2025.1524781 PMC11832717

[34] Fei F, Zhang D, Yang Z, Wang S, Wang X, Wu Z, Wu Q, Zhang S, The number of polyploid giant cancer cells and epithelial-mesenchymal transition-related proteins are associated with invasion and metastasis in human breast cancer, J. Exp. Clin. Cancer Res 34 (2015) 158. 26702618 10.1186/s13046-015-0277-8 PMC4690326

[35] Adams DL, Adams DK, Alpaugh RK, Cristofanilli M, Martin SS, Chumsri S, Tang CM, Marks JR, Circulating cancer-associated macrophage-like cells differentiate malignant breast cancer and benign breast conditions, Cancer Epidemiol. Biomarkers Prev 25 (2016) 1037–1042. 27197300 10.1158/1055-9965.EPI-15-1221 PMC4930681

[36] Carter SL, Cibulskis K, Helman E, McKenna A, Shen H, Zack T, Laird PW, Onofrio RC, Winckler W, Weir BA, Beroukhim R, Pellman D, Levine DA, Lander ES, Meyerson M, Getz G, Absolute quantification of somatic DNA alterations in human cancer, Nat. Biotechnol 30 (2012) 413–421. 22544022 10.1038/nbt.2203 PMC4383288

[37] Al-Sumidaie AM, Leinster SJ, Hart CA, Green CD, McCarthy K, Particles with properties of retroviruses in monocytes from patients with breast cancer, Lancet 1 (1988) 5–9. 2447453 10.1016/s0140-6736(88)90998-1

[38] Hamada T, Mohle R, Hesselgesser J, Hoxie J, Nachman RL, Moore MA, Rafii S, Transendothelial migration of megakaryocytes in response to stromal cell-derived factor 1 (SDF-1) enhances platelet formation, J. Exp. Med 188 (1998) 539–548. 9687531 10.1084/jem.188.3.539 PMC2212480

[39] Schachtner H, Calaminus SD, Sinclair A, Monypenny J, Blundell MP, Leon C, Holyoake TL, Thrasher AJ, Michie AM, Vukovic M, Gachet C, Jones GE, Thomas SG, Watson SP, Machesky LM, Megakaryocytes assemble podosomes that degrade matrix and protrude through basement membrane, Blood 121 (2013) 2542–2552. 23305739 10.1182/blood-2012-07-443457

[40] Huang H, Cantor AB, Common features of megakaryocytes and hematopoietic stem cells: what’s the connection? J. Cell. Biochem 107 (2009) 857–864. 19492306 10.1002/jcb.22184 PMC2741141

[41] Clawson GA, Matters GL, Xin P, Imamura-Kawasawa Y, Du Z, Thiboutot DM, Helm KF, Neves RI, Abraham T, Macrophage-tumor cell fusions from peripheral blood of melanoma patients, PLoS One 10 (2015) e0134320. 26267609 10.1371/journal.pone.0134320 PMC4534457

[42] Athanasou NA, Wells CA, Quinn J, Ferguson DP, Heryet A, McGee JO, The origin and nature of stromal osteoclast-like multinucleated giant cells in breast carcinoma: implications for tumour osteolysis and macrophage biology, Br. J. Cancer 59 (1989) 491–498. 2713238 10.1038/bjc.1989.102 PMC2247156

[43] Adams DL, Stefansson S, Haudenschild C, Martin SS, Charpentier M, Chumsri S, Cristofanilli M, Tang CM, Alpaugh RK, Cytometric characterization of circulating tumor cells captured by microfiltration and their correlation to the cellsearch((R)) CTC test, cytometry, Part A : the journal of the International Society for Analytical Cytology 87 (2015) 137–144. 10.1002/cyto.a.22613 25515318

[44] Adams DL, Zhu P, Makarova OV, Martin SS, Charpentier M, Chumsri S, Li S, Amstutz P, Tang C-M, The systematic study of circulating tumor cell isolation using lithographic microfilters, RSC Adv. 4 (2014) 4334–4342. 10.1039/C3RA46839A PMC4299665 25614802

[45] Chinen LTD, Torres JA, Calsavara VF, Brito ABC, Silva VSE, Novello RGS, Fernandes TC, Decina A, Dachez R, Paterlini-Brechot P, Circulating polyploid giant cancer cells, a potential prognostic marker in patients with carcinoma, Int. J. Mol. Sci 25 (2024). 10.3390/ijms25189841 PMC11432346 39337327

[46] Pirrello A, Killingsworth M, Spring K, Rasko JEJ, Yeo D, Cancer-associated macrophage-like cells as a prognostic biomarker in solid tumors, J. Liq. Biopsy 6 (2024) 100275. 40027315 10.1016/j.jlb.2024.100275 PMC11863711

[47] Adams DL, Alpaugh RK, Martin SS, Charpentier M, Chumsri S, Cristofanilli M, Adams DK, Makarova OV, Zhu P, Li S, Tang C-M, Stefansson S, Precision microfilters as an all in one system for multiplex analysis of circulating tumor cells, RSC Adv. 6 (2016) 6405–6414. 29093811 10.1039/c5ra21524b PMC5662211

[48] Adams DL, Alpaugh RK, Tsai S, Tang CM, Stefansson S, Multi-phenotypic subtyping of circulating tumor cells using sequential fluorescent quenching and restaining, Sci. Rep 6 (2016) 33488. 27647345 10.1038/srep33488 PMC5028835

[49] Adams DL, Adams DK, He J, Kalhor N, Zhang M, Xu T, Gao H, Reuben JM, Qiao Y, Komaki R, Liao Z, Edelman MJ, Tang CM, Lin SH, Sequential tracking of PD-L1 expression and RAD50 induction in circulating tumor and stromal cells of lung cancer patients undergoing radiotherapy, Clin. Cancer Res 23 (2017) 5948–5958. 28679765 10.1158/1078-0432.CCR-17-0802

[50] Stefansson S, Adams DL, Erschler WB, Le H, Ho D, A cell transportation solution that preserves live circulating tumor cells in patient blood samples, BMC Cancer. 16:300 (2016). 27150191 10.1186/s12885-016-2330-1 PMC4858886

[51] Lu X, Lu X, Kang Y, Organ-specific enhancement of metastasis by spontaneous ploidy duplication and cell size enlargement, Cell Res. 20 (2010) 1012–1022. 20603645 10.1038/cr.2010.93 PMC2932852

[52] Manjunath Y, Mitchem JB, Suvilesh KN, Avella DM, Kimchi ET, Staveley-O’Carroll KF, Deroche CB, Pantel K, Li G, Kaifi JT, Circulating giant tumor-macrophage fusion cells are independent prognosticators in patients with NSCLC, J. Thorac. Oncol 15 (2020) 1460–1471. 32416323 10.1016/j.jtho.2020.04.034

