# Supplementary material for: Phenotyping and clinical utility of phagocytic polyploid giant cancer macrophages in blood
Source: Cancer Lett. Author manuscript; Available in PMC 2026 May 28. (PMC13218590; doi:10.1016/j.canlet.2025.218007)
Supplement: Appendix A. Supplementary data [file NIHMS2170973-supplement-Appendix_A__Supplementary_data.pdf]

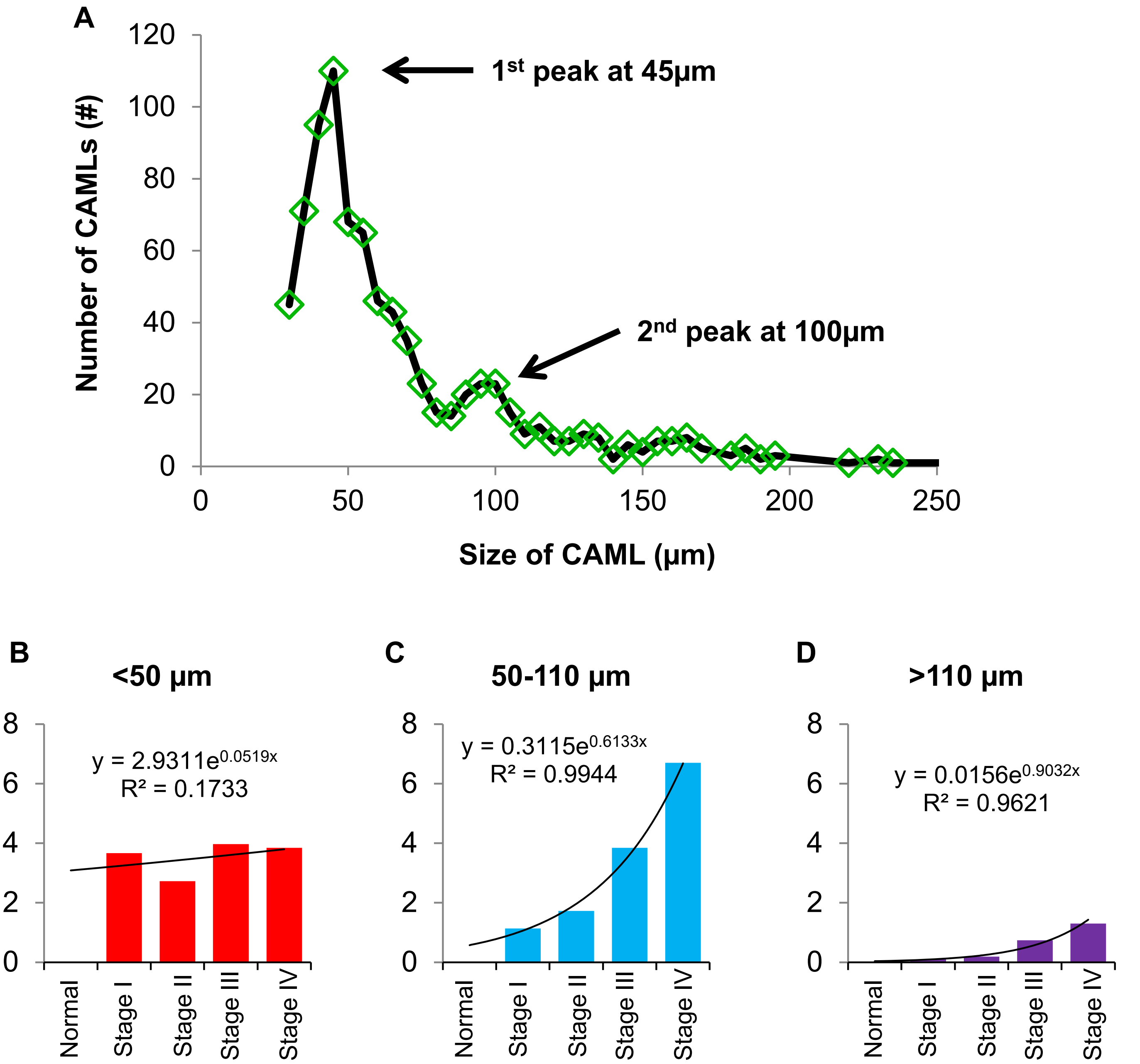

Figure S1. Bimodal distribution of CAML size and the modal relationships to stage. **A.** CAMLs (n=813) from patients (n=293) were measured based on the diameter of the cell. Number of CAMLs for each size were plotted and a bimodal pattern was observed, with peaks at 45µm and 100µm (black arrows). **B.** "Smaller" CAMLs with a size 30-49µm in diameter were found to be common in all stage s of disease but did not indicate disease progression (**Fig. 1**). **C.** Medium CAMLs with a size 50-110µm and **D.** Large CAMLs with a size of >110µm both exponentially correlated with stage of disease and both indicated progressive disease (**Fig 1**).

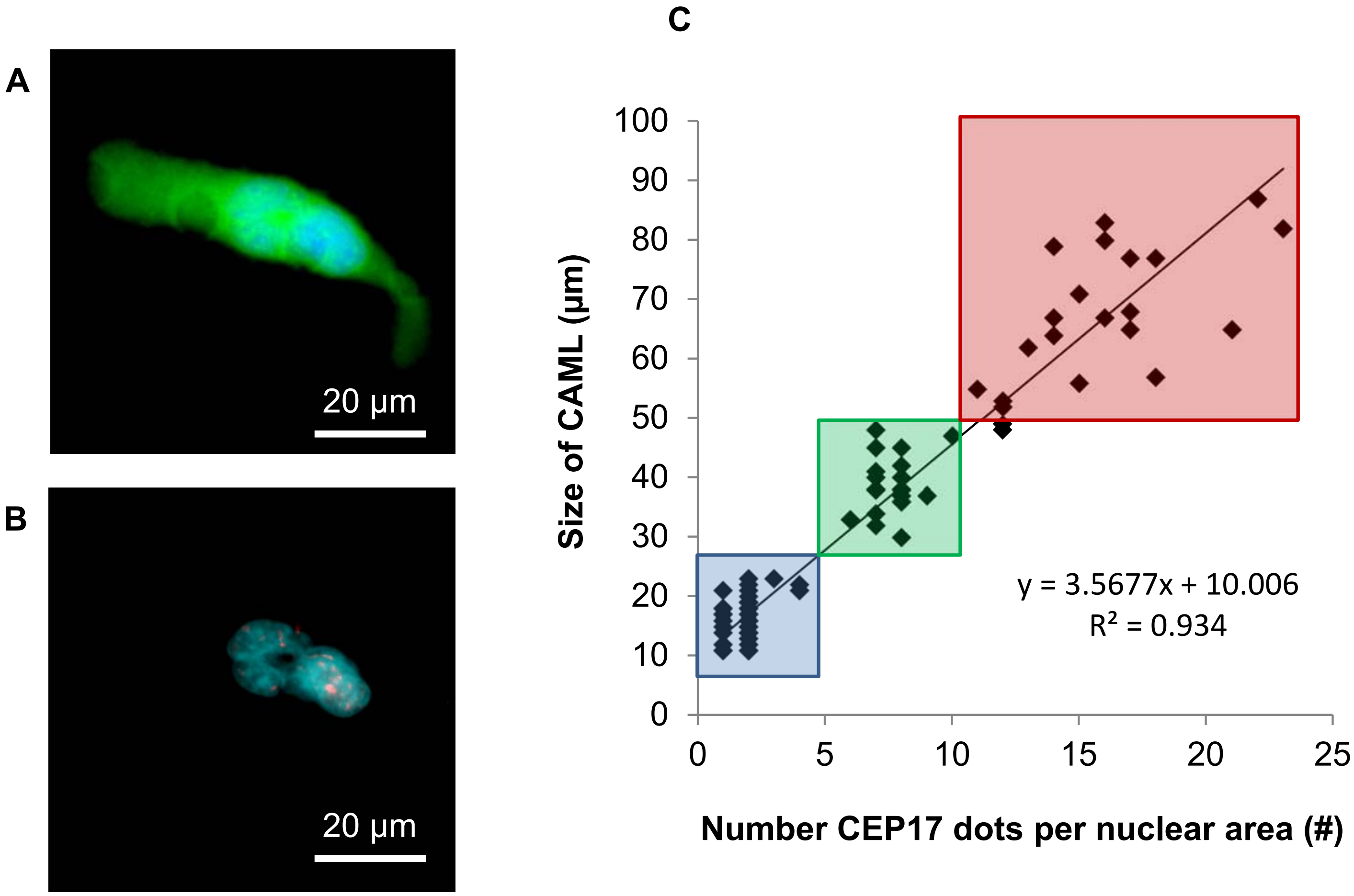

Figure S2. Comparing ploidy and CAML size. A. Example of a CAML from a breast cancer patient cytokeratin (green) and DAPI (blue). **B.** The CAML was then probed with DAPI (blue) and chromosome 17, CEP17 (Red dots), to determine ploidy of cell (16N). **C.** CAMLs and WBCs (n=108) were identified and measured by size using the cytoplasmic area of the primary antibody stains, described in *Materials and Methods,* and then probed with CEP17, as previously described (15, 38, 41). Ploidy, based on CEP17 number, was compared against the size of the corresponding cells. **C.** Ploidy status was highly correlated to cell size for both CAMLs and WBCs. Control WBCs had cell sizes of 8-22µm with ploidy distribution of 1N (16%), 2N (78%), 3N (2%) and 4N (4%), i.e. the blue shaded box. Smaller CAMLs ranging in size from 30-50µm had ploidy distribution of 6-8N (82%) and 9-12N (18%), i.e. the green shaded box. Larger CAMLs >50µm had a ploidy distribution of 11-12N (15%) and 13-26N (85%), i.e. the red shaded box.

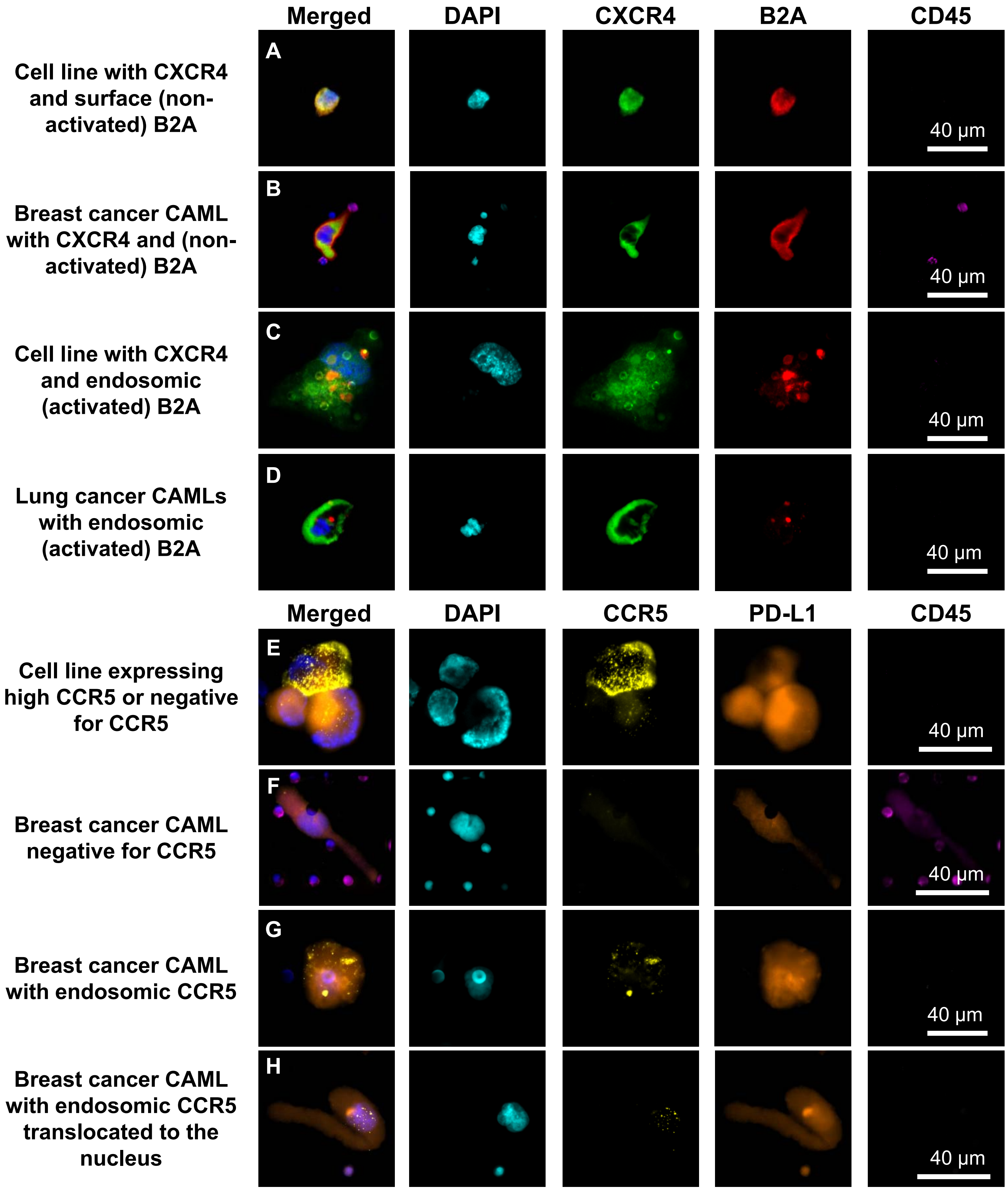

Figure S3. Inactive and activated CCR5 or Beta-2 Adrenergic receptor (B2A) on cells lines and in CAMLs from cancer patients. A. Cell line MB231 positive for CXCR4 and non-active surface receptor B2A. **B.** CAML positive for CXCR4 and non-active B2A. **C.** B2A on a MB231 cell activated with 25µM Isoproterenol (42-44), observed as endosomic vesicles. **D.** CAMLs with endosomic vesicles, indicating activation of B2A. **E.** Three MB231 cells are PD-L1+, top cell is surface positive and bottom 2 cells are low/negative for CCR5. PD-L1 was used as a positive marker. **F.** CAML found negative for CCR5. **G.** CAML with endosomic vesicles of CCR5 within the cell indicating activation. **H.** CAML with CCR5 endosomic vesicles translocated to the nucleus, indicating activation and perinuclear recycling of the CCR5 (42-45).

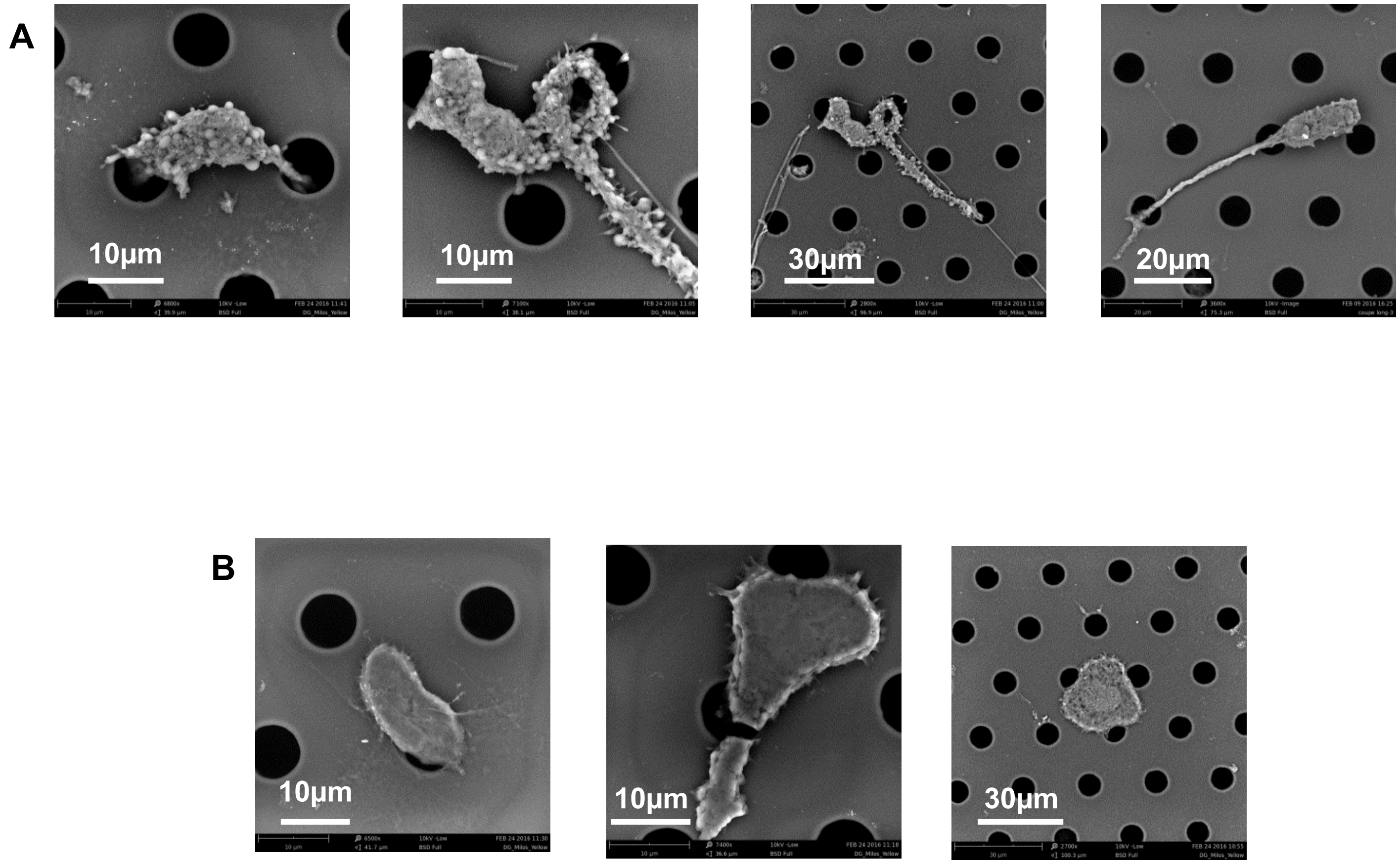

Figure S4. Additional SEM images of CAMLs blebbing or with pseudopodia-like protrusions. **A**. SEMs of CAMLs from breast or prostate cancer patients isolated on a microfilter with blebbing structures of various sizes pinching off from the CAML cell mass. **B.** SEMs of a CAMLs from breast or prostate cancer patients isolated on a microfilter with long (left panel) and short (center and right panels) protrusions, indicating active attachment to the filter substrate.

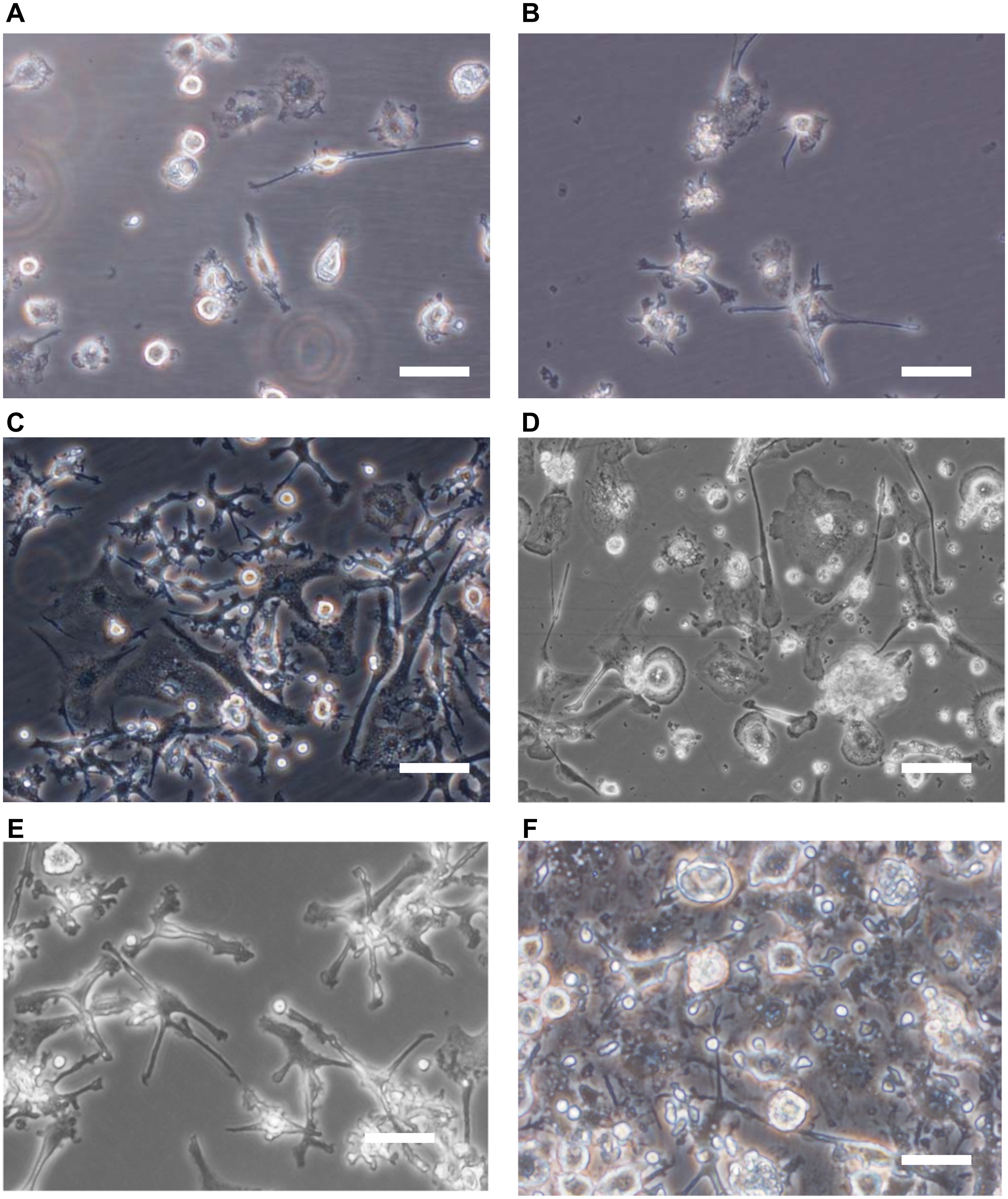

Figure S5. Additional figures of CAMLs culturing *ex vivo* in T flasks from low confluence (A and B) and high confluence (C, D, E and F). Cultures from 150 randomly-selected cultures (using computer-aided randomization) were plated to culture CAMLs (38-39, 46-47). 34 patients samples contained giant cells (22%) that proliferated which included Urogenital, head and neck, gastrointestinal (colorectal) , gynecological, lung, breast, and neuroendocrine tumors. All patients were anonymized and drawn into heparinized vacutainers collected at various cancer centers across the US. All cultured samples are assayed within 28 hours of collection. Blood sampled from anonymized healthy control volunteers (n=10) had no giant cells and did not produce any giant cells in culture. White Salce bars=200µm

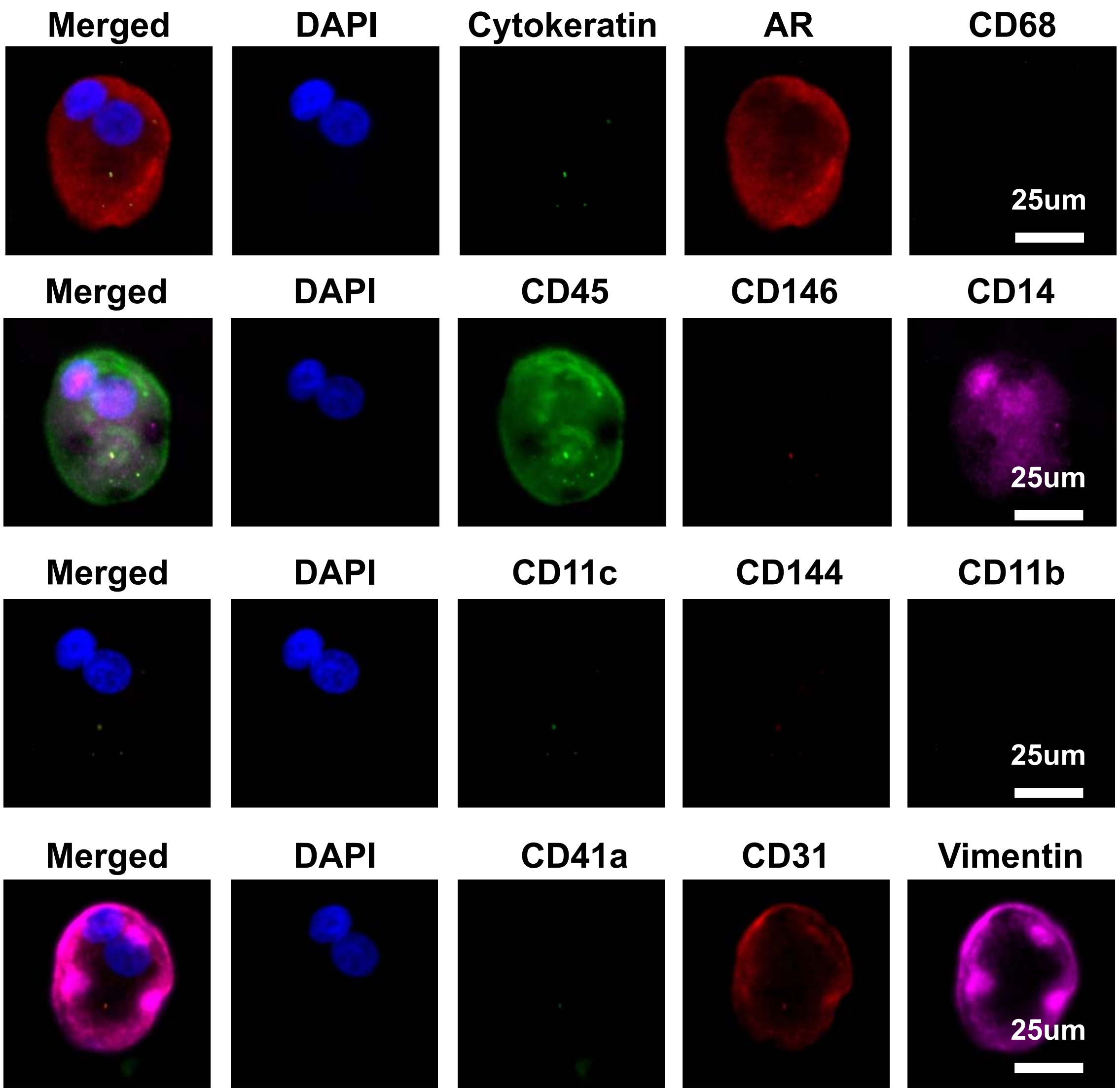

Figure S6. Staining of CAMLs after 6 weeks of *ex vivo* culture followed by QUAS-R for cell identification and subtyping. A. CAML cell culture from a prostate patient (**Fig. 4c-e**) was trypsinized from a culture plate and transferred to a filter for staining. CAMLs were subtyped by staining with prostate epithelial markers (cytokeratin and Androgen Receptor [AR]), white blood cell marker (CD45), macrophage markers (CD14, CD11b, CD11c and CD68), megakaryocyte marker (CD41a), angiogenic markers (CD146, CD144, CD31) and mesenchymal marker (Vimentin). After culture and ~50% plate confluency many of the original CAML phenotypic traits remained, including AR+, CD14+, CD31+, Vimentin+ and CD45+. In this case the CAML was cytokeratin negative. Square=60um

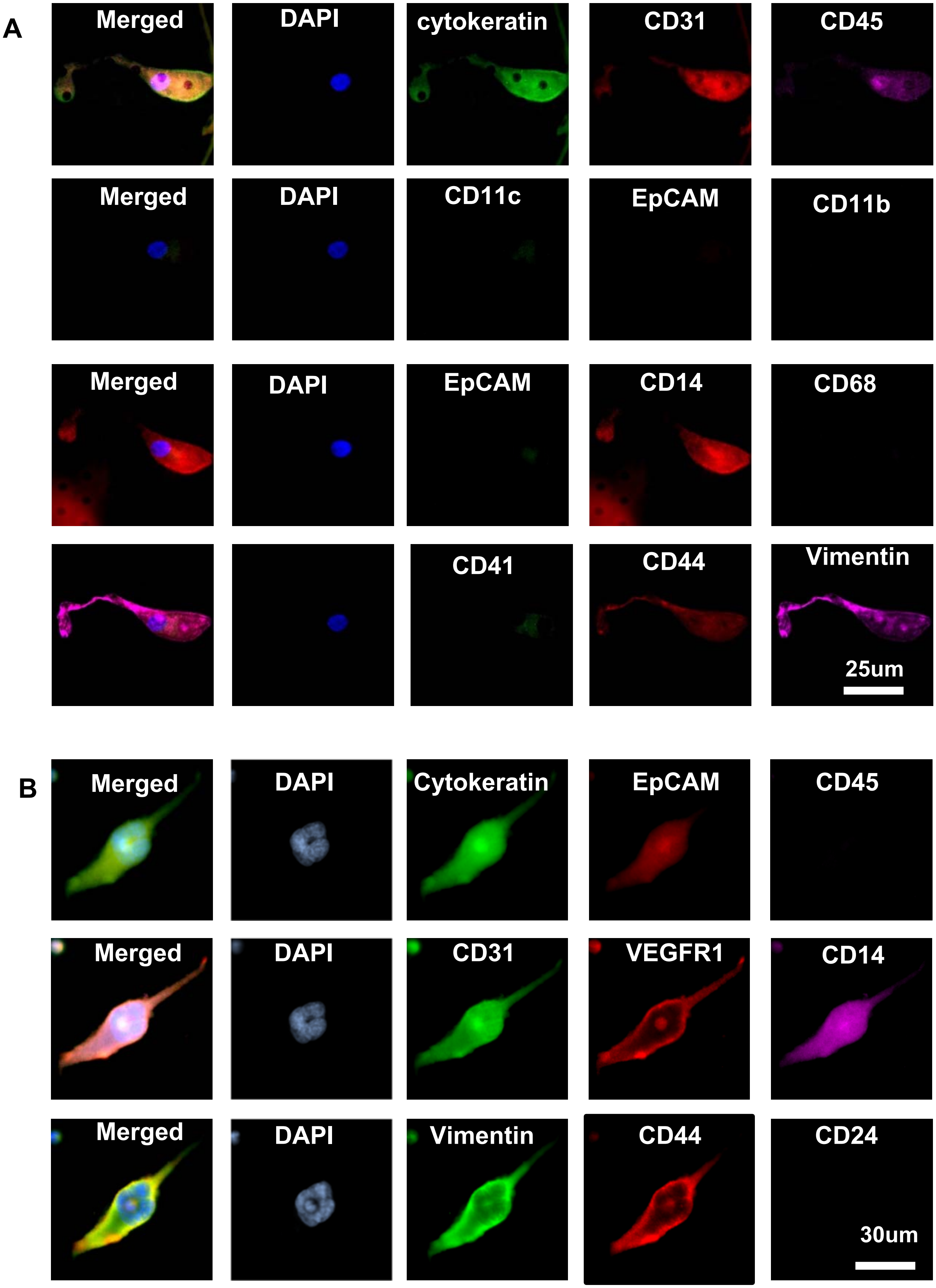

Figure S7. Additional Staining of key CAMLs biomarkers after 6 weeks of *ex vivo* culture followed by QUAS-R for cell identification and subtyping. A. CAML cell cultured from a metastatic colorectal patient. Cells were trypsinized from a culture plate and transferred to a filter for staining. **B**. CAML cell cultured from a metastatic Prostate patient. Cells were trypsinized from a culture plate and transferred to a filter for staining.

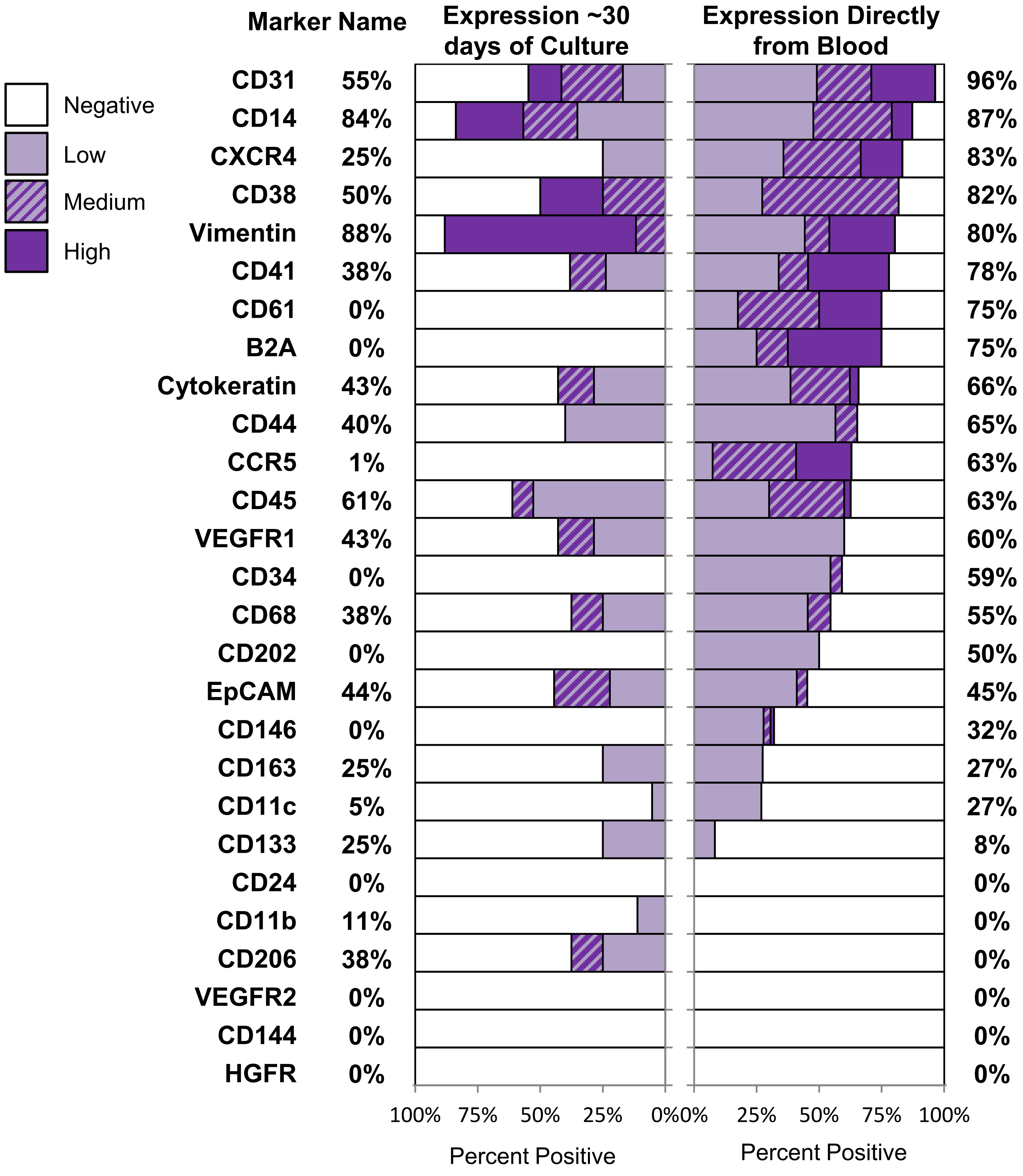

Figure S8. Staining of key biomarkers on CAMLs after 6 weeks of *ex vivo* culture versus staining of uncultured CAMLs isolated directly from patient blood. CAML staining of biomarkers from figure 3 is shown on the right bars. In parallel, CAMLs from patients were cultured and then stained with the same biomarker panels. Overall, most biomarkers absent in directly isolated CAMLs were also absent in cultured CAMLs, i.e. HGFR, CD144, CD24. However, multiple markers appeared to be reduced or disappear during culture, i.e. CD31, CXCR4, CD61, CD41, CD34 and CD146. Further, some makers appeared to increase during culture, i.e. Vimentin, CD206, CD11b.

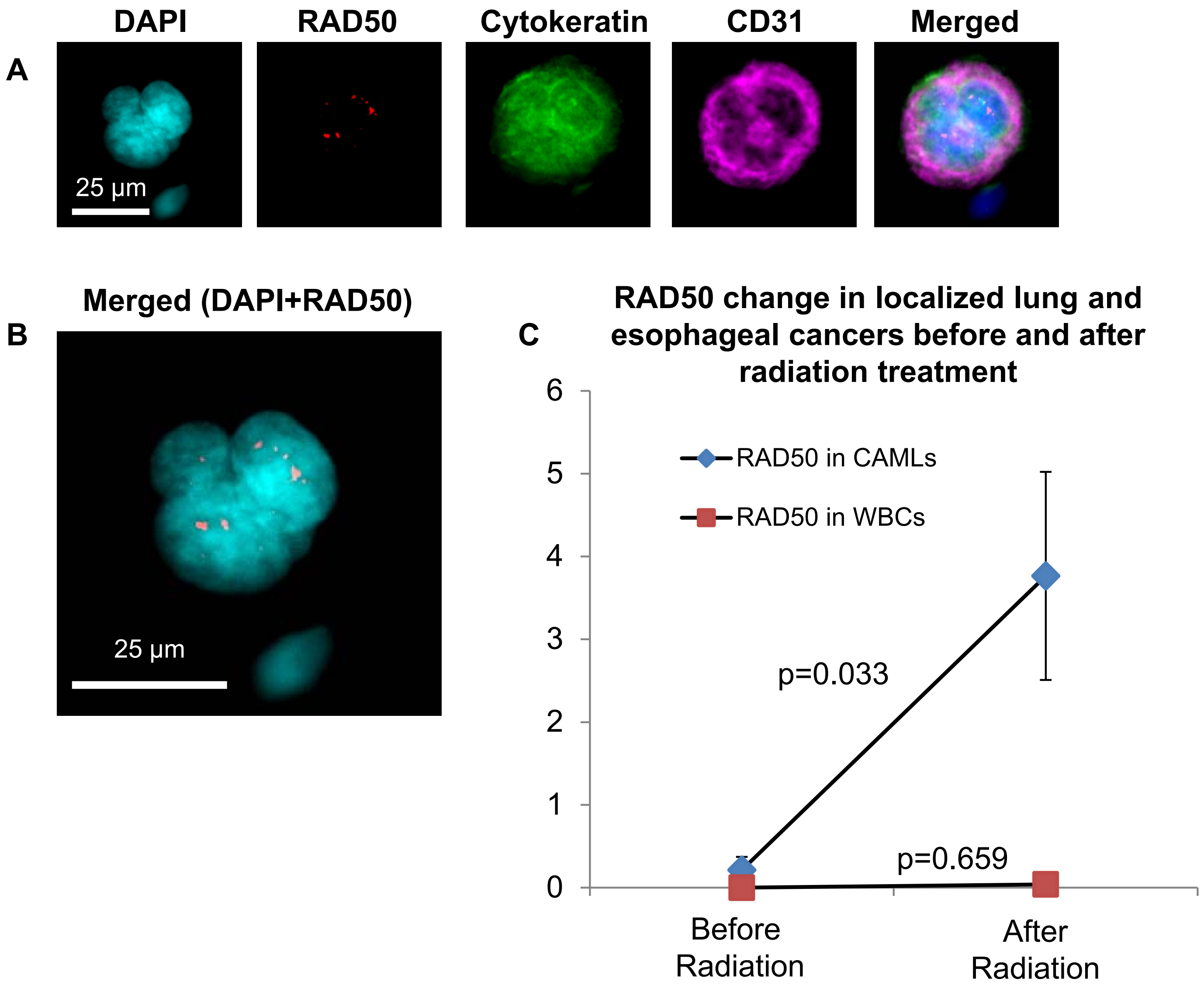

Figure S9. RAD50 foci formation after radiation exposure in localized lung (n=8) and esophageal (n=5) cancers. We have previously established that ionizing radiation induced foci (IRIF) labeled by RAD50 acts as a biological tag of cells directly exposed with targeted radiation at a primary tumor (41, 48). **A and B.** CAML from a lung cancer patient exposed to radiation with 8 foci of RAD50 (red) within the nuclear area (blue/DAPI). The CAML is also identified with Cytokeratin (green) and CD31 (violet). The formation of IRIF can be quantified by the number of RAD50 foci found in the nuclei which correspond to number of double stranded DNA breaks caused by the cell's direct exposure to radiation (40, 48). **C.** Tracking IRIF formation in CAMLs from patients (n=13) in localized (Stage I or II) disease indicates that the CAMLs originate from the treatment site, i.e. at the local primary tumor. CAMLs averaged 0.2 RAD50 foci per cell at baseline, prior to radiotherapy induction. After induction of directed radiotherapy to the primary tumor mass, CAMLs averaged 3.8 RAD50 foci per cell. White blood cells (WBCs) were used as controls for RAD50 foci with only 5 of 100 WBCs having any RAD50 foci.

Table S1. Patient population overview

		Number of patients (n=293)
Stage	I	62
	II	62
	IIIA	65
	IV	97
	unknown	7
Prior Chemotherapy		78
Prior Targeted Therapy		46
No prior therapy		169
Cancer subtype	Breast	59
	Esophageal	27
	Lung	59
	Pancreatic	59
	Prostate	52
	Renal Cell	37
Sex	M / F	157/136
Age	Median (Range min-max)	66 (27-92)

Table S2. Number at risk for Stage 1/2 by CAML size from Fig 1D

months	0	6	12	18	24
0-50μm	71	64	58	51	48
50-110μm	42	37	25	19	16
>110μm	11	8	5	4	2
50-110μm & >110μm	53	45	30	23	18

Table S3. Number at risk for Stage 3/4 by CAML size from Fig 1E

months	0	6	12	18	24
0-50μm	55	39	32	30	24
50-110μm	69	28	18	11	4
>110μm	38	11	8	5	4
50-110μm & >110μm	107	39	26	16	8

Table S4. Healthy Control population overview

		Number of patients (n=51)
Sex	M / F	26 / 25
Age	Median (Range min-max)	60 (32-81)

Table S5. Blood Processing Sites

Site name	Number pts recruited	Samples Filtered at site	Filters analyzed at site
Fox Chase Cancer Center	26	Yes	Yes
Mayo Cancer Clinic	39	Yes	No
Duke University	17	No	No
MD Anderson	86	No	No
Northwestern	28	Yes	Yes
OSHU	15	Yes	No
University of Maryland	24	Yes	Yes
University of Wisconsin	58	No	No
Creatv Core Facility	0	Yes (n=114)	Yes (n=114)

Table S6. CAML Phenotype and Number by Treatment and Sites of Metastases

	Number of pts	Average CAML Number (Range)	Average Max CAML Size (range)
Treatment Type			
Treatment Naive	149	3.57 (0-32)	65.75 (43-86)
Chemotherapy	92	17.34 (0-108)	60.44 (27-85
Hormone Therapy	27	10.37 (1-105)	64.82 (36-89)
Targeted Therapy	36	12.75 (0-105)	63.08 (27-92)
Site of metastasis			
Bone	44	15.30 (0-105)	96.55 (30-289)
Lung	31	12.94 (1-108)	84.19 (30-265)
Liver	30	10.63 (0-61)	85.60 (30-265)
Brain	9	16.70 (1-49)	106.11 (41-137)
Peritoneum	6	11.0 (2-27)	117.65 (38-389)
Adrenal	5	3.20 (0-7)	85.60 (0-265)
Other	21	15.14 (0-108)	83.62 (0-192
1 metastasis	62	8.45 (0-64)	74.45 (0-170)
>1 metastases	35	18.09 (1-108)	100.03 (30-289)

Table S7. Antibodies by manufacturer, concentrations used, positive controls used and negative controls used.

Antibody target	Supplier	Concentration	Positive control	Negative control
CD31	Miltenyi	5μg/mL	HUVEC	MCF-7
CD14	eBioscience	5μg/mL	HCC70	SUDHL1
CXCR4	eBioscience	10μg/mL	HeLa	PANC1
CD38	eBioscience	1.25μg/mL	MOLT-4	MB231
Vimentin	eBioscience	1μl/mL	HUVEC	MCF-7
CD41	eBioscience	10μl/mL	HEL	HUVEC
CD61	Biolegend	5μg/mL	CAKI-2	MCF-7
Beta-2-AR	Cell signaling	2.1μg/mL	MB-231	MCF7
Cytokeratin	Creatv Microtech	5μg/mL	KATOIII/MCF-7	HUVEC
CD44	eBioscience	1.25μg/mL	ES2/HeLa	LnCAP
CCR5	Novus	0.7μg/mL	MB231	MCF7
CD45	eBioscience	1.5μg/mL	MOLT4	HUVEC
VEGFR1	R&D systems	5μg/mL	HUVEC	MCF7
CD34	Biolegend	5μg/mL	HUVEC	MCF7
CD68	Biolegend	5μl/mL	THP1	BEWO/HUVEC
CD202	Biolegend	5μg/mL	MKN1	MB231
EpCAM	eBioscience	1.25μg/mL	MCF-7	HUVEC
CD146	eBioscience	5μg/mL	SKMEL28	MCF7
CD163	Biolegend	5μg/mL	SUDHL1	HCC70
CD11c	eBioscience	25μl/mL	THP-1	HUVEC
CD133	Miltenyi	20μl/mL	KATOIII/HT29	HUVEC
CD24	eBioscience	5μg/mL	MCF-7	MB-231
CD11b	eBioscience	25μl/mL	H441	HUVEC
CD206	Cell signaling	1.5μg/mL	MOLT4	MCF-7
VEGFR2	eBioscience	20μl/mL	H441	HUVEC
CD144	eBioscience	10μg/mL	HUVEC/MCF7	HeLa
HGFR	eBioscience	10μg/mL	HeLa	T47D
